# “Real-World” Evaluation of Lipid Oxidation Products and Trace Metals in French Fries From Two Chain Fast-Food Restaurants

**DOI:** 10.3389/fnut.2021.620952

**Published:** 2021-02-05

**Authors:** Adam Le Gresley, Gilbert Ampem, Simon De Mars, Martin Grootveld, Declan P. Naughton

**Affiliations:** ^1^Department of Chemistry and Pharmaceutical Sciences, Kingston University, Kingston-upon-Thames, United Kingdom; ^2^Leicester School of Pharmacy, De Montfort University, Leicester, United Kingdom

**Keywords:** aldehydes, legislated metals, lipid oxidation products, French fries, proton nuclear magnetic resonance, fast-food restaurants

## Abstract

Differences in lipid oxidation products (LOPs) and trace metal concentrations of French fry samples found between two global chain fast-food restaurants in the UK were investigated using high-resolution proton nuclear magnetic resonance (^1^H NMR) and inductively coupled plasma-optical emission spectrometry (ICP-OES) analyses, respectively, of extracts derived therefrom. Over the course of 3 days and 3 different diurnal time periods, samples of French fries (FFs) were analyzed, and comparisons of two different oil extraction methods were undertaken for the two restaurants involved. The magnitude of concentrations of LOPs extracted from FFs is discussed. Significant differences between 6/7 aldehyde classifications, and aluminum, manganese, vanadium, lead, iron, copper and nickel levels between samples from the two restaurants are also reported. Redox-active transition and further trace metal concentrations inversely correlated with FF oil sample LOP contents; this suggested an antioxidant rather than a pro-oxidant role for them.

## Introduction

The year 2020 marks the 160th anniversary of the birth of the British Fish and Chip Shop, reputed to have begun with the opening of the first such establishment by Joseph Malin in London in 1860. Whilst the process of frying can be traced back to Greece in the fifth Century BC, and was evidenced in the Roman cookbook by Apicus (Pullum Frontonianum, 400 AD), the chemical intricacies of the process have only come to light during the last 10 years or so. High-temperature frying episodes trigger a complex series of oxidation and polymerization reactions, leading to a multifarious mixture of products which add to the taste, aroma and texture of fried foods. LOPs formed from this thermally-induced peroxidation of frying oil unsaturated fatty acids (UFAs) have been shown to exert both mutagenic and carcinogenic properties (1, 2). They also have potent pro-inflammatory actions (3, 4), increase systolic blood pressure (5), and can also induce cell damage and death through the induction of chromosomal abberations (6, 7).

The impact of the frying process on vegetable oils which penetrate fried foods has been the subject of several recent reports, which have detailed the identification and determination of toxic aldehydes therein. However, these studies seldom consider “real-world” samples of foods which are readily available for human consumption, especially in Western diets. Consequently, this investigation is focused on the extraction of frying oils from French fries (FFs) cooked by two different chain restaurants, and the evaluation of frying oil and lipid oxidation product (LOPs) uptake by this commonly consumed fried food. This investigation was performed over several days and at different diurnal time-points, a critical criterion that has not been considered in previous studies (7–12). Equally, the levels of trace, redox-active trace metals were also determined in these sample in view of their well-known abilities to catalyze the lipid peroxidation process, and hence promote the adverse generation of LOPs.

This investigation considers the following three enquiries, which served as major objectives of the study:

1. What are the contents and patterns of secondary aldehydic LOPs in FF samples collected from fast-food chain restaurants, and is there a difference between the concentrations of different LOPs and catalytic redox-active metals found in these fried foods between two global chain restaurants?
2. Is there a pattern of FF-extracted LOPs which is related to their trace metal concentrations?
3. Can any inferences be drawn from any observed temporal variation in the FF contents of LOPs in this “real-world” scenario?

In light of the extensive consumption of FFs from fast-food restaurants, and the potential for high concentrations of potentially deleterious LOPs to develop over time with repeated culinary frying episodes, the effect of re-using oil and the impact of the day/time of sampling points was evaluated. High-resolution proton nuclear magnetic resonance (^1^H NMR) spectroscopy, which has already been recognized as an accurate and versatile analytical method, obviates the need for extensive purification of analytes prior to analysis (13, 14).

## Materials and Methods

### Chemicals

All chemicals were purchased from Sigma-Aldrich UK Ltd. (Gillingham, UK). The percentage purity of deuterated chloroform (C^2^HCl_3_), 1,3,5-tribromobenzene (TBB), and nitric acid (HNO_3_) were 99.5, 98, and 70%, respectively. Hydrogen peroxide (H_2_O_2_) was used as a 30% (w/v) aqueous solution. In addition, anhydrous sodium sulfate (Na_2_SO_4_), HPLC gradient-grade water (0.67 μS), and *n*-hexane were all analytical grade products.

### Collection of Fried Foods and Their Preparation for ^1^H NMR Analysis

Duplicate portions of FFs (103 ± 0.13 g XFF, and 113 ± 0.28 g YFF) were purchased at different times on different weekdays (Friday, Saturday, and Monday) from two local fast-food chain restaurants situated in London, UK. For confidentiality purposes, the restaurants featured are referred to as restaurants X and Y, and FF servings purchased therefrom are labeled as XFF and YFF, respectively. Collection days were Friday, 30 November 2018; Saturday, 01 December 2018; and Monday, 03 December 2018. Collection times on the above days were consistently in the morning, between 11 and 12 h; afternoon, between 14 and 15 h; and evening, between 19 and 20 h. Demands were higher on Friday and Saturday. As might be expected, in contrast, Monday had a lower demand. All samples were placed immediately into separate Ziploc plastic bags and stored at −20°C until ready for frying oil/LOPs extraction.

The rationale behind the collection days and times was to consider the potentially higher demand for FFs on Fridays and Saturdays, in the context of a lower demand for them on Mondays, and to evaluate the impact that this may have on the observed patterns of LOPs found therein.

**Figure 1:**
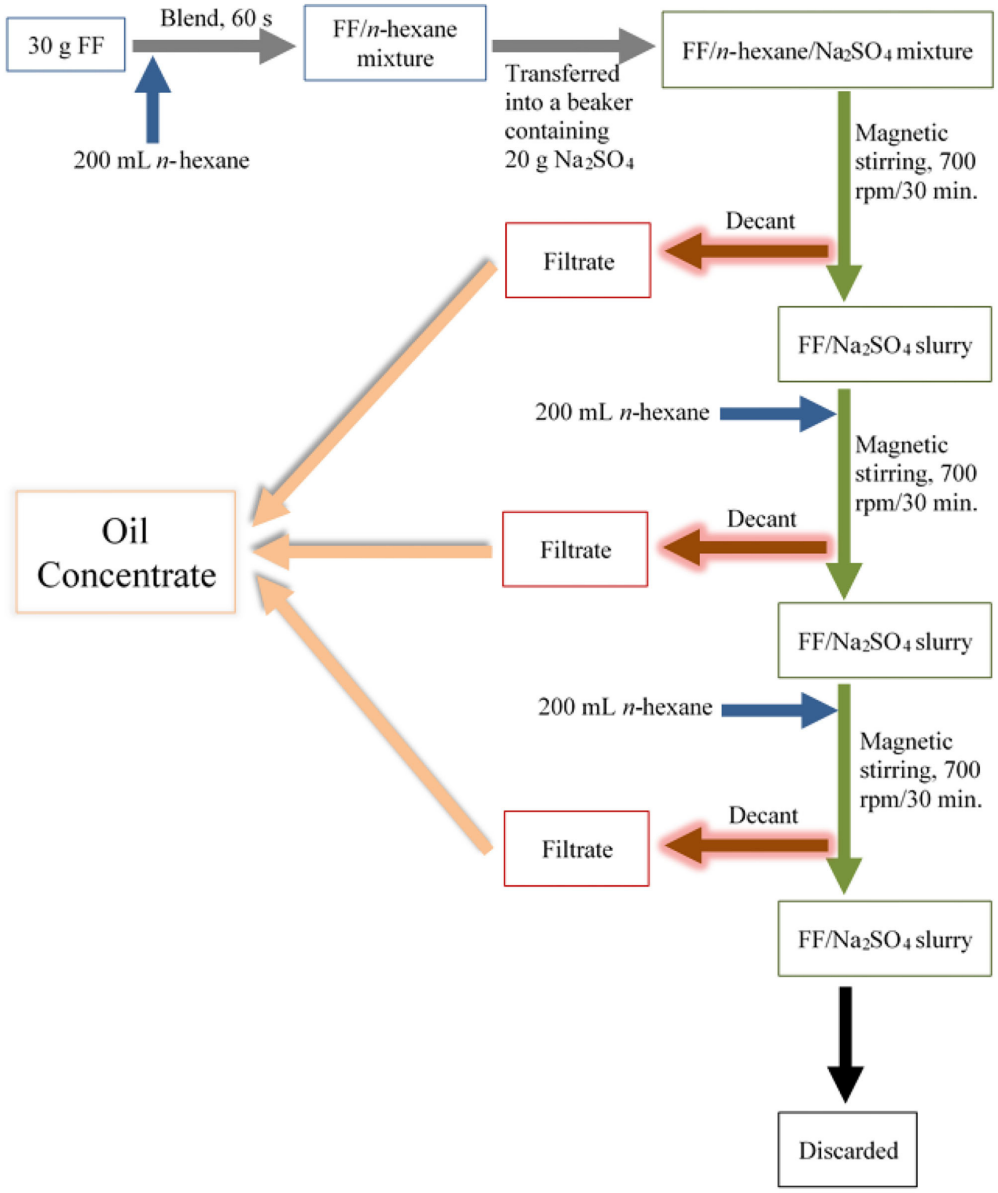
Stepwise process employed for the extraction of culinary oils and secondary aldehydic LOPs from FF samples purchased from restaurant chains X and Y. FF, French fries; rpm, revolutions per minute.

Culinary oils were extracted from the purchased FF samples from restaurants X and Y using *n*-hexane. In duplicate, FF samples (30 g) were cold-blended with a 200 mL volume of *n*-hexane in a 1.5 L Coline glass jug blender of dimensions 16 × 18 × 38 cm (Model CW 1298, China), and operated at 800 W for 60 s. This was then transferred to a 1,000 mL volume glass beaker containing 20 g anhydrous sodium sulfate (Na_2_SO_4_), after which the beaker was covered with aluminum foil. Anhydrous Na_2_SO_4_ served as an inert drying agent to absorb traces of water that may be present in the blended FF samples. Extraction of culinary oil from the blended FF/*n*-hexane/Na_2_SO_4_ mixture was then carried out on a Fisher Scientific magnetic stirrer (Model DLM 1895X4; Iowa, USA) operated at 700 rpm for a period of 30 min. The *n*-hexane supernatants, which contained oil extracts from the FFs, were removed by careful decantation into clean, air-dried beakers. The residual blended FF/Na_2_SO_4_ slurries were then further extracted 2-fold, each for 30 min., and each using 200 mL *n*-hexane at each stage (Figure 1). Samples of *n*-hexane oil extracts from XFF and YFF samples are shown in the supporting information (Supplementary Figure 1).

Overall, culinary oil was extracted from 30 g masses of FFs three times using a total of 600 mL *n*-hexane, hence the ratio of 1 g FF to 20 mL *n*-hexane. *n*-Hexane-oil filtrates from the repeated extractions were pooled together, and the solvent *n*-hexane evaporated in a fume hood leaving the extracted culinary oil to concentrate in the beaker. Oil yield was calculated using equation 1. All extracted culinary oils were transferred to 5 mL glass vials and stored at −20°C until further analysis (Supporting Information Proton NMR Measurements to Analysis of Trace Metals).

(1) $$\begin{array}{l} \text{Oil yield}\left(\text{\%}\right)=\frac{\text{Weight of oil}}{\text{Weight of FF}}\times 100 \end{array}$$

This extraction technique was compared with the method originally developed by Moumtaz et al. (12) but slightly modified in this study. Accurately, 5.0 g of FF was mechanically homogenized using a mortar and a pestle. This was transferred into a Thermo Scientific Nunc 50 mL conical centrifuge tube to which was then added 5.0 mL deuterated chloroform (or deuterochloroform) (C^2^HCl_3_) and tightly sealed. The mass ratio of FF to C^2^HCl_3_ was predominantly 1:1 (g:mL). The homogenates in conical centrifuge tubes were placed on a Stuart Orbital Shaker (Model SSL1, UK) operated at 175 rpm for 1 h. These were then transferred into a Thermo Scientific Heraeus Labofuge 400R Centrifuge (Germany) operated at a 4,500 relative centrifugal force (rcf) level at 4°C for a period of 20 min. Following that, 0.50 mL of the clear supernatant was removed and then treated with a 0.1 mL 1,3,5-tribromobenzene (TBB) (final concentration in NMR tube is 1.10 × 10^−^3 M), and this was subjected to a ^1^H NMR determinations as detailed in the Supporting Information Proton NMR Measurements to Analysis of Lipid Oxidation Products. In retrospect, all C^2^HCl_3_ extractions were performed in duplicate for every FF portion purchased from restaurants X and Y, alongside duplicated *n*-hexane extractions. A discussion of the two extraction methods is available under section Analytical Comparisons of Methods Utilized to Extract Aldehydes From FF Samples. “Analytical comparisons of methods utilized to extract aldehydes from FF samples” and in the Supporting Information Deuterated Chloroform and n-Hexane Extraction Techniques, Characterization of LOPs, Supplementary Table 6.

### Analysis of Trace Metals

#### Microwave Digestion

All culinary oils obtained for this study, including those from FFs, were analyzed for their trace metals' compositions. This part of the study aimed to determine the effect that available trace metals may have on the evolution of LOPs.

The method for digesting culinary oil was adopted from Nunes et al. (15) and modified. The digester employed for the breakdown of the oils was Microwave Accelerated Reaction System (MARS) (North Carolina, USA), Model MARS 240/50 with specifications: 220/240 volts, 15 A maximum current, 50 Hz frequency, 3,100 W maximum power, and a 1,600 W maximum microwave power. The digester was also equipped with a 40-batch capacity 55 mL MARSXpress trifluoromethoxyl-polytetrafluoroethylene (TFM-PTFE) vessels. Accurately, 0.5 g of FF oil extract was weighed into the TFM-PTFE vessels, to which was added 3.5 mL concentrated nitric acid (70% HNO_3_) and 1.0 mL of hydrogen peroxide (>30% w/v H_2_O_2_). The TFM-PTFE vessels were tightly sealed and capped and then placed in the microwave oven. Digestion of the test samples was performed in seven phases under varying time and microwave power at constant pressure (55 Bar) and temperature (240°C) (Supporting Information Analysis of Trace Metals, Supplementary Table 1). All the digests were clear solutions, and these were transferred to 15 mL glass vials and further diluted with a 5.5 mL dilute HNO_3_ (1.0% (v/v) prepared in high-performance liquid chromatography (HPLC) gradient grade water of 0.67 μS conductivity). The resulting solutions were filtered, and the filtrate subjected under an inductively coupled plasma-optical emission spectroscopy (ICP-OES) analysis. Reagent blanks were prepared following the same protocol used for the test samples. The experiment was conducted in duplicate for each test sample and reagent blank.

As a precaution, all glassware and TFM-PTFE vessels used in this study were thoroughly washed and rinsed with HPLC gradient-grade water, after which they were rinsed again with concentrated nitric acid (70% HNO_3_) and dried. Specifically, 7.0 mL volumes of 70% (v/v) HNO_3_ was added to each TFM-PTFE vessel, and they were further cleaned using the same microwave operating program. These precautionary measures were ensured prior to the digestion of each batch of oil (16).

#### ICP-OES Determination of Trace Metals

Inductively Coupled Plasma Optical Emission Spectrometry (ICP-OES), which is also referred to as Inductively Coupled Plasma Atomic Emission Spectrometry (ICP-AES), was employed as a technique to investigate the role that trace metals may play in catalyzing the evolution of LOPs. The ICP-OES instrument, ULTIMA-2C Horiba JY ISA (France), was used for the determination of the analytes Aluminum (Al), Arsenic (As), Barium (Ba), Cadmium (Cd), Cobalt (Co), Chromium (Cr), Copper (Cu), Iron (Fe), Manganese (Mn), Molybdenum (Mo), Nickel (Ni), Lead (Pb), Titanium (Ti), Thallium (Tl), Vanadium (V), and Zinc (Zn). Among these analytes, Cd, Co, Cr, Cu, Fe, Mn, Mo, Ni, Ti, V, and Zn are transition metals. Non-transition metals analyzed in this study are Al, Pb, and Tl. Furthermore, according to the International Olive Oil Council (IOC) (17), As, Cu, Fe, and Pb are classified as legislated metals, highlighting the practical importance of critically regulating their levels. The operating conditions of the ICP-OES instrument are described in supporting information (Supplementary Table 1) for quantitative analysis. The calibration standard solutions for the above analytes were prepared from a multi-element standard solution (16). Calibration curves built for Al, As, Cd, Co, Cr, Cu, Fe, Mn, Ni, Pb, Ti, Tl, and Zn were within the 0–50,000 μg.kg^−1^ range. That of Ba and Mo were within 0–10,000 μg.kg^−1^, and V was within the 0–100,000 μg.kg^−1^ range. All measurements were conducted in duplicate, and the final concentrations determined were expressed as mean ± S.D. values. In general, As, Co and Mo were undetectable in the studied oils, and hence their absence from Tables 4, 7, Supplementary Table 7.

#### ^1^H NMR Analysis

All NMR experiments were undertaken on a Bruker Avance III 600MHz Instrument equipped with a TXI probehead. Details of pulse sequences and the general experimental methodology performed are available in the Supporting Information Proton NMR Measurements.

#### Statistical Analysis of FF Sample Aldehydic LOP and Trace Metal Ion Datasets

Primarily, univariate ANOVA was conducted on firstly acylglycerol fatty acid classification contents (and IVs), and secondly the seven aldehydic LOPs monitored. For this purpose, a hierarchal experimental design (model 1) was employed, and this incorporated “Between-Restaurants,” “Between-Sampling Days-nested-Within-Restaurants,” and “Between-Sampling Time-Points-nested-Within-Sampling Days” effects as the main sources of variation considered. Since sampling days and sampling time-points were pre-selected, these were all treated as fixed and not random effects. Further effects considered were all first-order interactions, specifically the Restaurants × Sampling Days, Restaurants × Sampling Time-Points, and Sampling Days × Sampling Time-Points effects in order to explore the significance of any differential, non-additive patterns of ^1^H NMR analyte responses observed. ANOVA was performed using Addinsoft, *XLSTAT2014* and *2020* software modules.

In a second ANOVA-based model (model 2), the statistical significance of Between-Restaurant and Between-Extraction Technique differences between the mean values of each aldehydic LOP classification concentration in FF samples were univariately determined (Addinsoft, *XLSTAT2014* and *2020*). Aldehyde levels were analyzed as total FA content-normalized values to allow for volume and method differences between the two extraction techniques employed.

For model 2, the overall ANOVA-based experimental design for this investigation was classified as a 3-factor system with the “Between-Extractions,” “Between-Restaurants,” and “Between-Aldehydes” factors involved being fixed effects at 2, 2, and 7 classification levels, respectively. Hence, this ANOVA model comprised the 3 main fixed effect factors, their 3 associated first-order interactions, and fundamental error.

For univariate comparisons of the mean trace metal contents of *n*-hexane-extracted FF oil samples between restaurants X and Y, false discovery rate (FDR)-corrected two-sample *t*-tests were performed. A multivariate (MV) comparison of these datasets was also conducted using partial least squares-discriminatory analysis (PLS-DA) using *MetaboAnalyst 5.0* (2020) software. Data were generalized logarithmically (glog)-transformed and autoscaled prior to analysis (autoscaling represents subtraction of column variable means and division by their respective standard deviations for all trace metal data entries).

Moreover, a model for the prediction of FF oil extract aldehydic LOP concentrations from their corresponding trace metal levels (12 in total) was constructed using a principal component regression (PCR) strategy. For this purpose, a maximum of 5 principal components (PCs) from the trace metal dataset was considered (data were again glog-transformed and autoscaled before analysis).

## Results

### Characterization of FF and Oil Extracts

#### Total Lipid Acylglycerol Content

**Figure 2:**
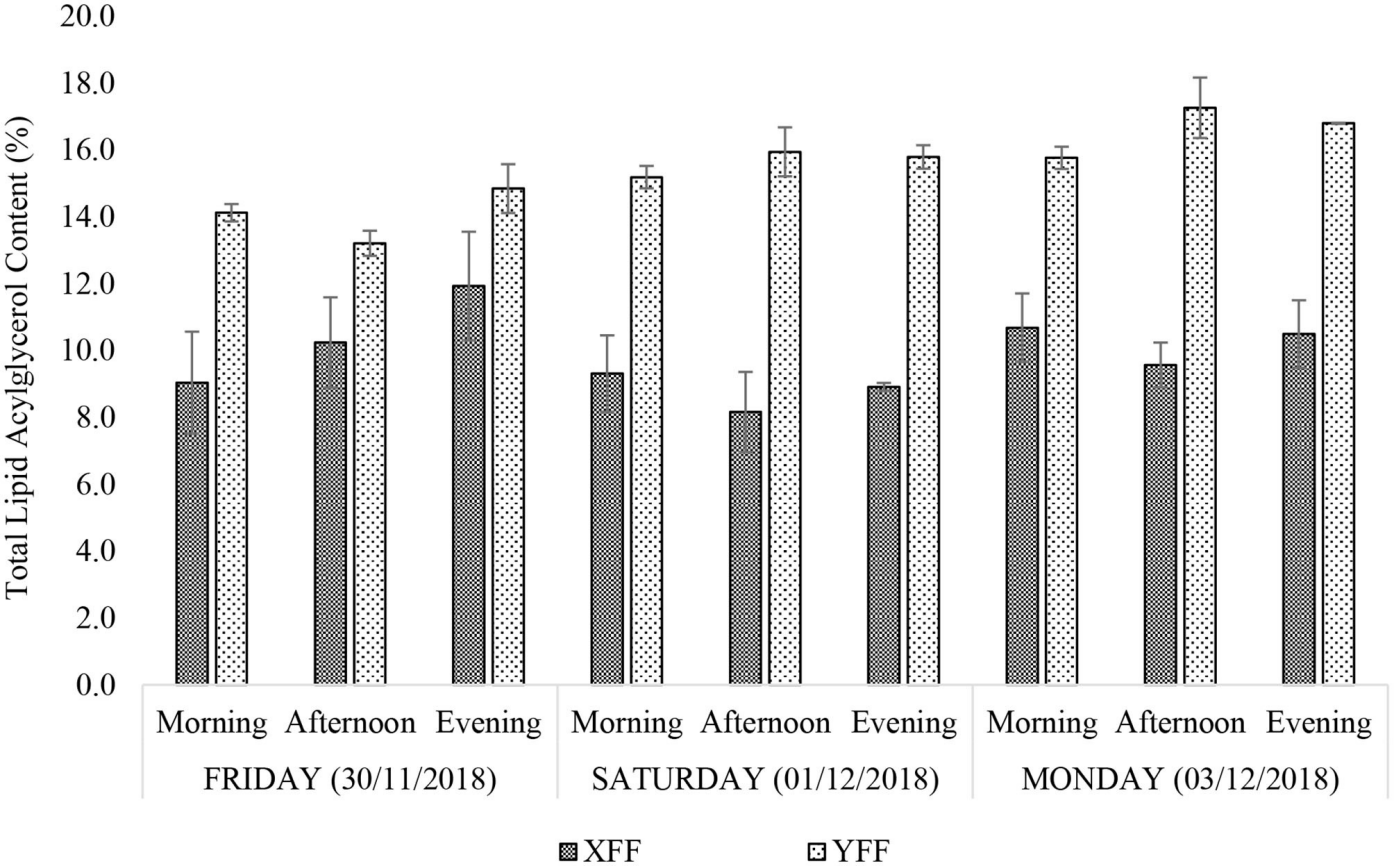
Percentage (w/w) total lipid acylglycerol contents of FF samples purchased from restaurants X and Y. XFF and YFF, French fries sampled from restaurants X and Y, respectively. All values are presented as mean ± SD values.

When expressed as a percentage of mass, the total lipid acylglycerol contents extracted from the FF samples obtained from fast-food restaurants X and Y varied across the days with little intra-day variation (Figure 2). Restaurant Y showed consistently higher total lipid acylglycerol contents [13.2–17.3% (w/w)] than restaurant X [8.2–11.9% (w/w)]. Amongst the sampling times and days, total FF lipid acylglycerol contents for restaurant X was least on Saturday (01/122018) afternoon and simultaneously highest on Friday (30/11/2018) evening. Restaurant Y yielded the least and the highest total FF lipid acylglycerol contents on Friday (30/11/2018) and Monday (03/12/2018) afternoons, respectively. The differences in total lipid acylglycerol contents between FF portions collected from restaurants X and Y may be attributed to the frying oils employed, cultivar differences in potatoes, differences in frying practices (temperature, duration and technique of frying, etc.), and the physical dimensions of the raw or uncooked FFs. Pre-frying practices such as washing and soaking of “raw” FFs, and the duration that these may have spent in contact with water and/or salt (or other additives), may also contribute to variations in total lipid acylglycerol contents recorded for the FF samples.

#### Acyl Groups

##### Major Acyl Groups

**Figure 3:**
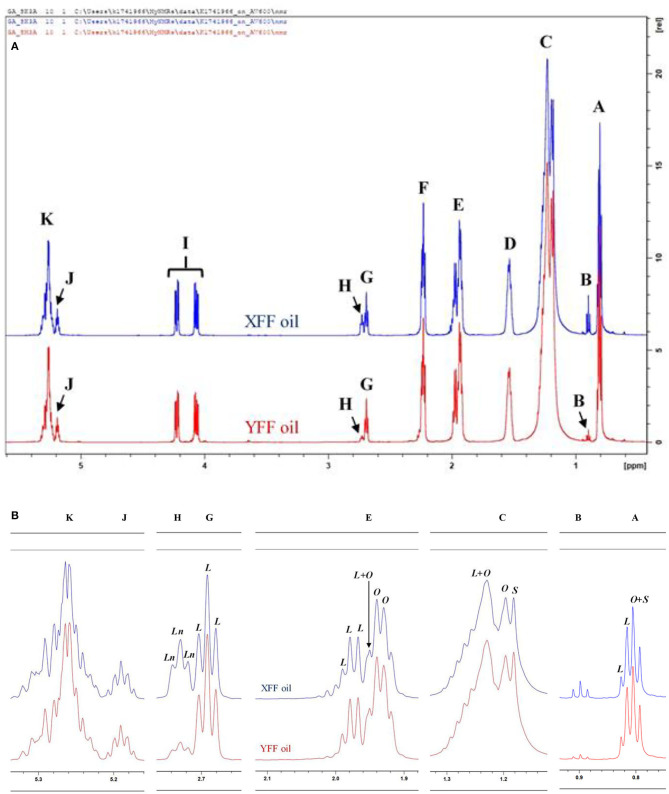
^1^H NMR spectra of frying oils collected from restaurants X and Y, showing **(A)** major acylglycerol functions, and **(B)** expanded regions of resonances A, B, C, E, G, H, J, and K present within the 0.0–5.4 ppm regions of *n*-hexane-oil extracts of FF samples purchased from restaurants X and Y. XFF and YFF, Restaurant X and Y French fries, respectively; *Ln*, Linolenoylglycerol signal; *L*, Linoleoylglycerol signal; *O*, oleoylglycerol signal; *S*, saturated fatty acid acylglycerol signal. Letter assignments of resonances correspond to those provided in Table 1. ^1^H NMR spectra of frying oils collected from restaurants X and Y, showing **(A)** major acylglycerol functions, and **(B)** expanded regions of resonances A, B, C, E, G, H, J, and K present within the 0.0–5.4 ppm regions of *n*-hexane-oil extracts of FF servings purchased from restaurants X and Y. XFF and YFF, Restaurant X and Y French fries, respectively; *Ln*, Linolenoylglycerol signal; *L*, Linoleoylglycerol signal; *O*, oleoylglycerol signal; *S*, saturated fatty acid acylglycerol signal. Letter assignments of signal resonances (A, B, C, E, G, H, J, and K) correspond to those provided in Table 1.

Figures 3A,B show the ^1^H NMR spectral profiles of C^2^HCl_3_-reconstituted *n*-hexane extracts of typical XFF and YFF samples, respectively. All equations used in generating the molar concentrations are delineated in Supporting Information Methods. Extracts from XFF and YFF samples were strikingly similar based on their ^1^H NMR profiles. Slight variations were the relative intensities of signals B and G. The chemical shifts, multiplicities, and chemical function assignments of these ^1^H NMR signals are outlined in Table 1. The general assignment of these signals was made according to previously published data available (18, 19).

**Table 1:** Assignments of bulk, intense ^1^H NMR signals of acylglycerol functions present in the ^1^H NMR profiles of XFF and YFF oil samples, including their chemical shift values and multiplicities.

			**Functional group**	
**Signal**	**Chemical shift (ppm)**	**Multiplicity**	**Condensed group**	**Classification**
A	0.739–0.869	*t*	–C**H**_3_	Saturated, oleic, and linoleic acyl groups
B	0.869–0.928	*t*	–C**H**_3_	Unsaturated ω-3 acyl groups
C	1.105–1.347	*t*	–(C**H**_2_)*n*–	Bulk chain acyl groups
D	1.487–1.598	*m*	–OCO–CH_2_-C**H**_2_-	Acyl groups except for those of DHA, EPA, and ARA
E	1.876–2.026	*m*	–C**H**_2_-CH-CH–	Acyl groups except for the –C**H**_2_- of DHA function β- to the carbonyl group
F	2.172–2.304	*dt*	–OCO–C**H**_2_-	Acyl groups except for those of DHA
G	2.653–2.713	*dd*	-HC–C**H**_2_-CH-	Diunsaturated fatty acid ω-6 acyl groups
H	2.713–2.758	*dd*	-HC–C**H**_2_-CH-	Triunsaturated ω-3 acyl groups
I	4.014–4.282	*dd, dd*	–C**H**_2_OCOR	Glyceryl backbone functions
J	5.157–5.216	*m*	>C**H**OCOR	Glyceryl backbone functions
K	5.216–5.340	*m*	–C**H**-C**H**–	Acyl chain olefinic functions

*d, doublet; t, triplet; m, multiplet; dd, doublet of doublets; dt, doublet of triplets; (ω-3), omega-3 fatty acid functions; ω-6, omega-6 fatty acids; DHA, docosahexaenoylglycerol acyl groups; EPA, eicosapentaenoylglycerol acyl groups; ARA, arachidonoylglycerol acyl groups. Letter assignments and characteristics correspond to those provided in Figure 3 and in Supplementary Figure 5*.

Signal A (0.739–0.869 ppm) is a triplet assignable to the terminal methyl group protons (–C**H**_3_) of all saturated, oleic, and linoleic acyl groups (Figure 3A). When expanded, a composite oleic acid and saturated FA signal (*O*+*S*) was dominant over that of linoleic acid (*L*) (Figure 3B). Signal B (0.869–0.928 ppm) is a triplet arising from the terminal–C**H**_3_ function of ω-3 acyl chains. Comparatively, the intensity of signal B was higher in XFF oil samples (Figures 3A,B). Chemical shift differences between signals A and B lies in the proximity of their –C**H**_3_ acyl groups to the double bond of the acyl chain. Signal C is ascribable to the bulk methylene protons [–(C**H**_2_)_*n*_–] of all acyl groups, and Signal D represents the –OCO–CH_2_-C**H**_2_- methylene protons, and is typically present in all acyl groups except for docosahexaenoic acid (DHA), eicosapentaenoic acid (EPA) and arachidonic acid (ARA) acyl groups. Signal C comprises an overlapped *L*+*O*, O and S signal resonances, where those of *O* and *S* were both of similar albeit lower intensities (Figure 3B).

Signal E is *mono*-allylic protons (–C**H**_2_-CH-CH–) of all unsaturated groups, except for the –C**H**_2_- acyl group in β-position of DHA (Table 1, Figure 3A). When expanded, signal E was dominated by a composite of *L, O* and *L*+*O* resonances (Figure 3B). Signal F, at 2.172–2.304 ppm, is ascribable to the –OCO–C**H**_2_- protons of all acyl groups except for those of DHA. Both the G and H resonances are multiplets (apparent but not triplets) arising from the *bis*-allylic proton (-HC–C**H**_2_-CH-) functions of PUFAs, i.e., linoleic and linolenic acid acyl groups, respectively (Figures 3A,B). Signal I is attributable to the protons on carbon atoms 1 and 3 of the glyceryl backbone (–C**H**_2_OCOR), and signal J located at δ = 5.157–5.216 ppm arises from its >C**H**OCOR proton environment. Signal K, a multiplet within the 5.216–5.340 ppm chemical shift range, is assignable to the olefinic protons (–C**H**-C**H**–) of all oil unsaturated FAs (Table 1, Figures 3A,B). Of course, olefinic proton ^1^H NMR intensities determine the iodine value of culinary oils. Hence, the greater the intensity of signal K, the larger the iodine value, and therefore the greater the unsaturation degree of oils analyzed.

Overall, although there were no clearly distinct differences between XFF and YFF oils regarding the major acyl function resonances characterized in Figure 3, although small differences in the intensities of some of their resonances facilitated differentiation between them. According to informal discussions with the managers in restaurants X and Y, sunflower oil and rapeseed oil were the culinary oils used in frying FFs. This may be correct for restaurant X in view of the presence of signal B (unsaturated ω-3 acyl groups) and H (triunsaturated ω-3 acyl groups), which may only arise from linolenoylglycerol-containing rapeseed oil. In contrast, sunflower oil does not contain signal B and H. The presence of both signals B and H in YFF oil, however, may suggest a blend of culinary oils that contain omega-3 and linolenic acid-containing acyl groups (Table 1, Figure 3).

##### Molar Percentage Contents of Major Acyl Chains

**Figure 4:**
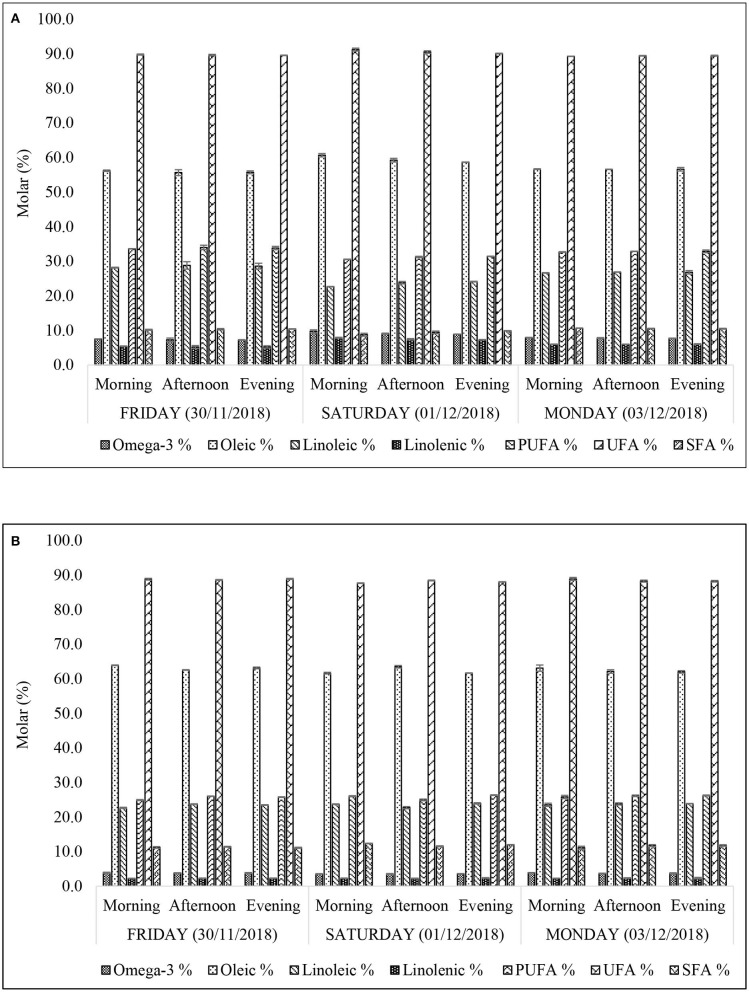
Concentrations of acylglycerol FA groups of *n*-hexane-extracts frying oils from FF samples purchased from restaurants X **(A)** and Y **(B)**. All values are presented as mean ± SD values.

The ^1^H NMR-determined acyl groups (both key and derived ones) were in the order: UFA (89.3–91.3% XFF; 87.7–88.9% (w/w) YFF) > oleic (oleoylglycerols) (55.7–60.7% XFF; 63.1–63.9% (w/w) YFF) > PUFA (30.6–34.0% XFF; 24.9–26.3% (w/w) YFF) > linoleic (linoleoylglycerols) (22.7–28.8% XFF; 22.7–24.0% (w/w) YFF) > SFA (8.8–10.7% XFF; 11.1–12.3% (w/w) YFF) > omega-3 (ω-3) acylglycerols (7.3–9.8% XFF; 3.5–3.9% YFF) > linolenic (linolenoylglycerols) (5.2–7.9% XFF; 2.2–2.4% (w/w) YFF) (Figures 4A,B). Total ω-3 and linolenoylglycerol acyl group levels in XFF oil extracts were at least 2- and 2–3-fold higher than those of the YFF samples. The percentage distributions of the acyl groups appeared to be quite stable across the sampling time-points. Nonetheless, it was observed that the culinary oil used for frying FFs obtained from restaurant X may have been changed after the Friday evening (30/11/2018) work shift (Figure 4A). This was reflected by the major acyl chains of XFF oil extracts obtained on Friday evening (30/11/2018) and Saturday morning (01/12/2018). Indeed, there was a sharp increase in total UFAs (89.6 to 91.2%), oleoylglycerols (55.7 to 60.9%), total ω-3 acylglycerols (7.3 to 9.7%), and linolenoylglycerols (5.2 to 7.9%), whilst simultaneously recording a sharp decrease in total PUFAs (33.8 to 30.6%) > linoleoylglycerols (28.6 to 22.7%) > SFAs (10.4 to 8.8%) in this order of magnitude (Figure 4A). This observation appears to be consistent with a change of frying oil from an oxidatively-degraded to a fresh (unused) form, since the FAs most susceptible to oxidative deterioration (ω-3 acylglycerols) are replenished somewhat. Therefore, the regular and careful monitoring, recording and replacement of thermo-oxidized culinary oils by fast-food restaurants will serve to limit the amounts of LOPs therein, which evolve in a time-and use-dependent manner.

##### Iodine Value

**Figure 5:**
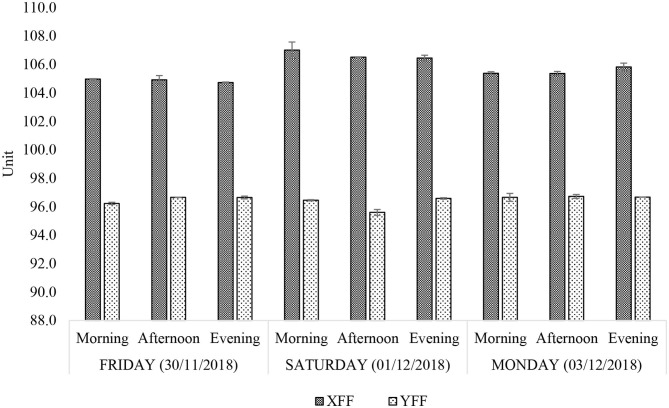
Iodine value (unit) of *n*-hexane-extracted culinary frying oils from FF samples purchased from restaurants X and Y. All values are presented as mean ± SD values.

Iodine value (IV) serves as an index of the unsaturation degree of an oil. Hypothetically, it is the number of grams of iodine absorbed by a 100 g quantity of fat or oil under prescribed experimental conditions. According to Figure 5, the IV of XFF and YFF sample oil extracts ranged between 104.7–107.0 and 95.5–96.7 units, respectively. The larger the IV of an oil, the higher the number of unsaturated >C=C < units present, and these values are consistent with the higher levels of total UFAs and PUFAs present in the XFF samples, as noted above. Oil extracts of YFF samples, according to their IV, therefore do appear to be slightly more resistant to oxidation, although it should be noted that the PUFA contents of both these FF-extracted oils would be expected to be already depleted somewhat in view of their prior exposure to high-temperature frying episodes. Like the acyl group FA content determinations (Figure 4), the IV levels of both XFF- and YFF-extracted oils did not vary across all sampling time-points. However, a considerably higher increase in IV on Saturday morning (31/11/2018) is an indication that restaurant X changed their oil following the Friday evening (30/11/2018) work shift (Figure 5).

#### Minor Compounds Detectable

**Figure 6:**
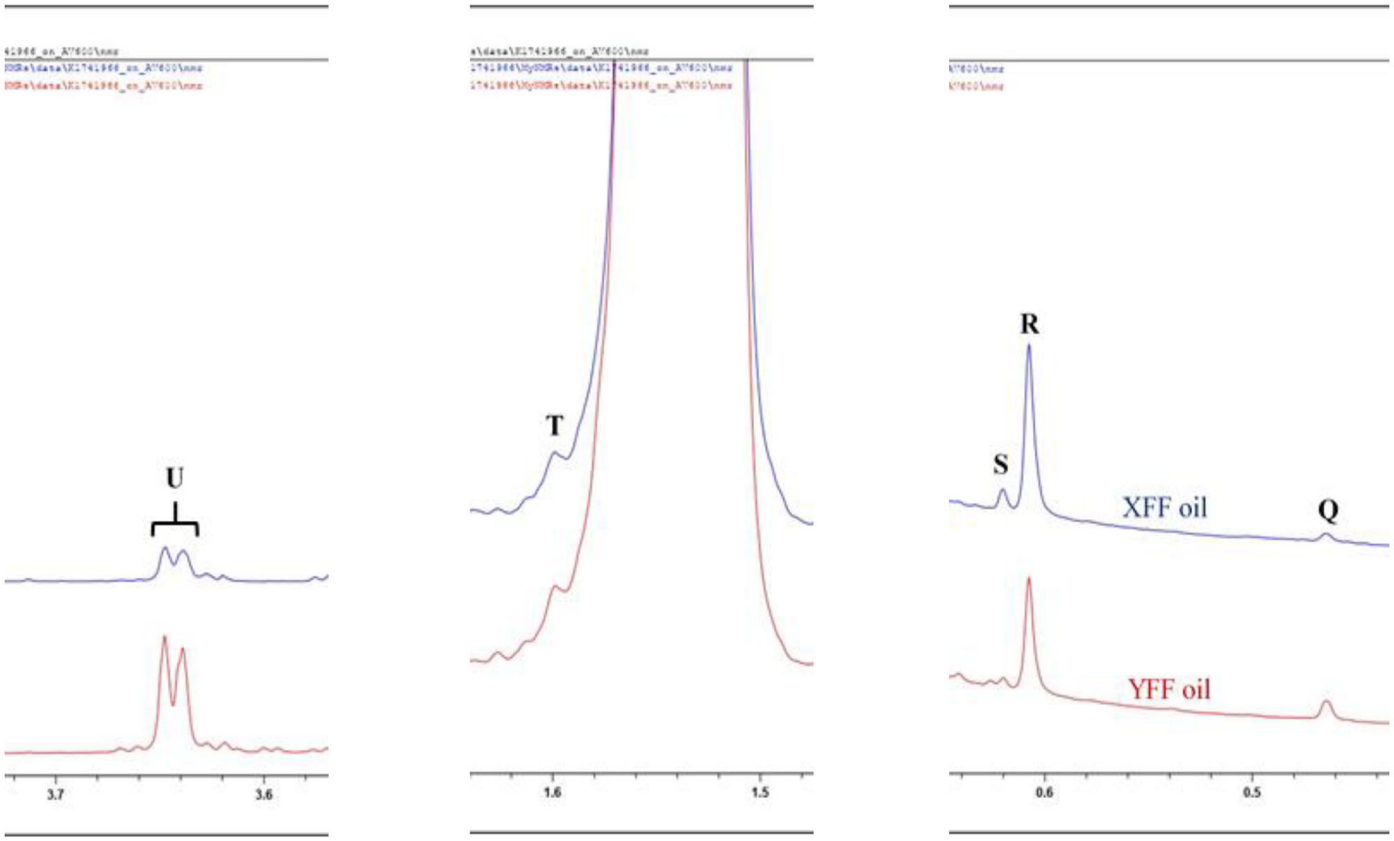
^1^H NMR spectra of frying oils collected from restaurants X and Y, showing minor compounds present with resonances within the 0.0–3.7 ppm regions of *n*-hexane-oil extracts of FFs purchased from restaurants X and Y. XFF and YFF, Restaurant X and Y French fries, respectively. Letter assignments of resonances correspond to those provided in Table 2.

Minor compounds identified and characterized included sterols [Δ7-avenasterol, β-sitosterol and Δ5-campesterol (and/or cholesterol), Δ5-stigmasterol and brassicasterol] and squalene, as well as 1,2-diacylglycerols (Table 2, Figure 6). The general assignment of these signals was made according to a previously published report (19).

**Table 2:** Assignment of the ^1^H NMR signals of minor compounds present in the ^1^H NMR profiles of XFF and YFF oils (sterols, stanols, and 1,2-diacylglycerol hydrolysis products), including chemical shift values, multiplicities, and their associated functional group assignments.

			**Functional group**	
**Signal**	**Chemical shift (ppm)**	**Multiplicity**	**Condensed group**	**Classification**
Q	0.457–0.472	*s*	–C**H**_3_ (C-18)	Δ7-Avenasterol
R	0.595–0.615	*s*	–C**H**_3_ (C-18)	β-Sitosterol, Δ5-Campesterol (or C-18 C**H**_3_ Cholesterol)
S	0.615–0.623	*s*	–C**H**_3_ (C-18)	Δ5-Stigmasterol and Brassicasterol
T	1.594–1.610	*s*	Terminal –C**H**_3_	Squalene
U	3.630–3.657	*d*	–C**H**_2_OH	1,2-Diacylglycerols

*s, singlet; d, doublet. Letter assignments and characteristics correspond to those provided in Figure 6 and in Supplementary Figure 6*.

Signal R may be ascribable to β-sitosterol, Δ5-campesterol or cholesterol. Although cholesterol may be present at low levels in vegetable oils, the processing of animal products is a common practice at commercial restaurants, and therefore cross-contamination of vegetable oils with animal lipids may elevate its corresponding signal intensity in ^1^H NMR spectra acquired on used vegetable oils.

Squalene (signal R) is a triterpene which serves as an important precursor for the biochemical synthesis of all sterols, regardless of its plant or animal origin (Figure 6).

1,2-Diacylglycerols (signal U) are naturally present in oils at low concentrations, and arise from triacylglycerol hydrolysis, along with 1.3-diacylglycerols and 1(3)- or 2-monoacylglycerols. Thermo-oxidation of the oils, however, may lead to the hydrolysis of triacylglycerols to 1,2-diacylglycerols, and hence differences observed in their resonance intensities in the XFF and YFF oil extracts may reflect this (Figure 3A). ^1^H NMR profiles showed that FFs purchased on Friday (31/11/2019) showed a higher signal intensity of 1,2-diacylglycerols in XFF oil extract than in YFF one. In contrast, YFF oil extracted from FFs purchased on Saturday (01/12/2019) and Monday (03/12/2019) had a higher signal intensity of 1,2-diacylglycerol resonances than those of XFF oils from samples purchased on same days.

Whilst these minor components are in invariably present at low to trace concentrations, the sterols and stanols detectable potentially offer health-friendly benefits to humans in view of their cholesterol-blocking activities *in vivo* (20–22).

**Figure 7:**
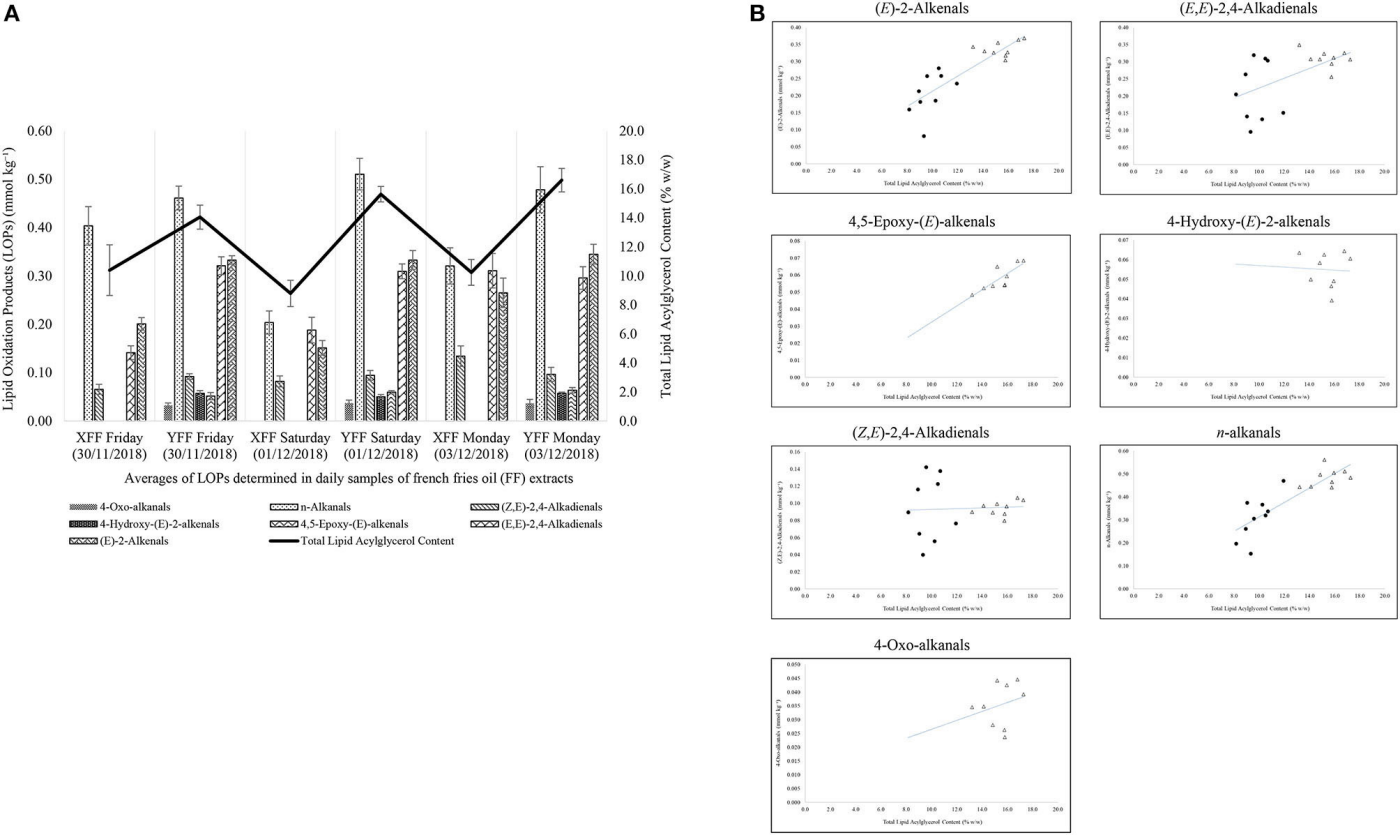
**(A)** Secondary aldehydic LOPs concentrations of FF samples collected from restaurants X and Y on consecutive weekly days (Friday, Saturday, and Sunday), and their total lipid contents. These data reveal a significant correlation between aldehyde and total lipid levels. All values are presented as mean ± SD values. **(B)** Relationships between mean FF concentrations of aldehydic LOPs and total lipid contents of FF samples for restaurants X (filled spheres) and Y (unfilled triangles).

According to Figure 7A, it is evident that restaurant X staff changed their oil throughout the monitoring period. This resulted in a sharp drop in LOPs on Saturday (01/12/2018). In addition, the total lipid acylglycerol content was directly proportional to the levels of LOPs quantified. Greater total lipid acylglycerol contents resulted in greater amounts of NMR-detectable LOPs. When shown in a scatter plot (Figure 7B), the individual aldehydic LOPs quantified at each sampling time-point had a unique positive correlation with the total lipid acylglycerol content of the oils extracted in FF purchased from restaurant X and Y. Accordingly, coefficient of determination (R^2^) values for the correlations between (*E*)-2-alkenals, (*E,E*)-2,4-alkadienals, 4,5-epoxy-(*E*)-alkenals, 4-hydroxy-(*E*)-2-alkenals, (*Z,E*)-2,4-alkadienals, *n*-alkanals and 4-oxo-alkanals and total acylglycerol content extracted from the mornings, afternoons and evenings of the sampling days were 0.73, 0.31, 0.66, 0.003, 0.003, 0.73, and 0.07 respectively (assuming linear relationships), i.e., strong correlations for the most highly concentrated aldehydes [(*E*)-2-alkenals, 4,5-epoxy-(*E*)-alkenals, and *n*-alkanals], but not so for (*E,E*)-2,4-alkadienals, 4-hydroxy-(*E*)-2-alkenals, (*Z,E*)-2,4-alkadienals, and 4-oxo-alkanals (Figure 7B).

### Characterization of LOPs

The thermo-oxidation of culinary oils leads to the productions of LOPs. LOPs either belong to the primary or secondary classes. All LOPs identified in XFF and YFF oil extracts are profiled in Supplementary Figures 2–4 and their respective signal resonances are defined in Supplementary Tables 2–4.

### Analytical Comparisons of Methods Utilized to Extract Aldehydes From FF Samples

For the analysis of FF sample aldehyde content datasets, we primarily normalized all these to total extracted acylglycrols therein in order to overcome differences in the levels of oil extracted per sample between the two methods employed, and also differing extraction volumes and techniques utilized (Supplementary Table 6). Indeed, *n*-hexane extract involved the analysis of ‘neat' oils exhaustively extracted from FF samples (Figure 2, Supplementary Figure 1), whereas the C^2^HCl_3_ extraction process involved the direct extraction of *ca*. 1 g masses of FFs, which contained 8–17% (w/w) total lipid contents (Supplementary Figure 12).

**Figure 8:**
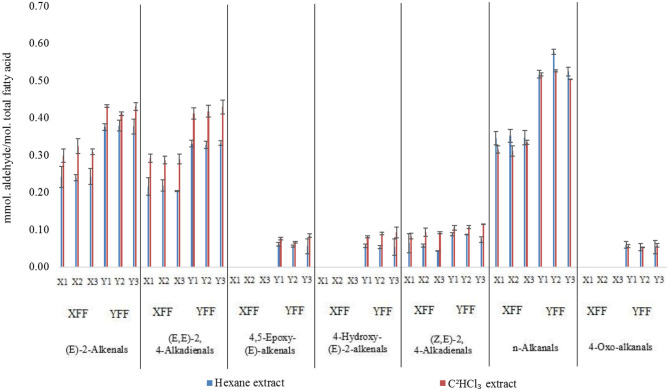
Aldehyde contents (mmol. aldehyde/mol. total fatty acid) of FFs normalized to total extracted acylglycerols for deuterochloroform (C^2^HCl_3_) and exhaustive *n*-hexane methods employed for the ^1^H NMR analyses of FF samples collected from restaurants X and Y. All values are presented as mean ± SD values. Data presented are consistent with those in Supplementary Table 6.

Figure 8 shows a bar diagram plot of all aldehydes determined using both these extraction techniques, and generally it is clear that despite some small differences observed, overall, there was a reasonable level of similarity between aldehyde levels determined by these two different methods. However, it appears that for at least some of the aldehydes analyzed, the C^2^HCl_3_ extracts yielded marginally higher levels than those determined via the *n*-hexane extraction process. For example, this was the case for the most predominant (*E*)-2-alkenal, (*E,E*)- and (*Z,E)*-2,4-alkadienals classifications (*p* < 10^−6^, ANOVA model 2), but not so for *n*-alkanals, nor for all other aldehyde classes monitored. These differences, albeit small ones, are presumably explicable by small losses of relatively volatile α,β-unsaturated aldehydes occurring during the evaporative stages during the *n*-hexane extractions. Such aldehydes are likely to include (*E*)-2-octenal [boiling point (b.pt) 84–86°C] and (*E,E*)-2,4-decadienal (b.pt 115°C), which are the most predominant (*E*)-2-alkenals and (*E,E*)-2,4-alkadienals derived from the peroxidation of culinary oil linoleoylglycerols. As expected, there was also a very highly significant difference observed between the restaurant sources of these FF samples for all aldehydes evaluated (*p* = < 10^−6^ to < 10^−4^), with the exception of (*Z,E)*-2,4-alkadienals.

ANOVA performed according to model 1 revealed highly significant “Between-Restaurant” differences between all ^1^H NMR-determined % acylglycerol classification contents and IV parameters investigated (Table 3A), and these are fully consistent with higher [MUFA]:[PUFA] concentration ratios for restaurant Y FF oil sample extracts over those of restaurant X, amongst other differences in their contents. Moreover, there were also significant differences “Between-Sampling Days-Within Restaurants” for all of these parameters, and also “Between-Sampling Time-Points-Within-Sampling Days” for four of the PUFA content classifications. Likewise, the Restaurants × Sampling Days interaction effect was significant for all these parameters, and this indicated that the pattern and/or extent of differences between the analyte responses varied significantly between the sampling days involved, as noted above. Additionally, the Restaurant × Sampling-Time-Points interaction effect was found to be significant for IVs only. As expected, the Sampling Days × Sampling Time-Points interaction effect was not significant for any of the analytes evaluated, and this indicates that, despite their being highly significant differences between sampling time-points for four of these parameters, their acylglycderol response patterns did not differ significantly within sampling days for each restaurant involved.

**Table 3A:** Statistical significance of “Between-Restaurants” (R), “Between-Sampling Days within Restaurants” (D), and “Between-Sampling Time-Points within Sampling Days” (T) sources of variation for % acylglycerol fatty acid contents and corresponding IVs according to the Model 1 ANOVA-based hierarchal experimental design.

	**Restaurants (R)**	**Days (D)**	**Time-points (T)**	**R × D**	**R × T**	**D × T**
Omega-3-acylglycerols	<10^−6^	<10^−6^	7.72 × 10^−3^	<10^−6^	ns	ns
Oleoylglycerols	<10^−6^	2.17 × 10^−5^	4.01 × 10^−2^	1.13 × 10^−6^	ns	ns
Linoleoylglycerols	<10^−6^	<10^−6^	1.79 × 10^−2^	<10^−6^	ns	ns
Linolenoylglycerols	<10^−6^	<10^−6^	ns	<10^−6^	ns	ns
Total PUFAs	<10^−6^	<10^−6^	1.51 × 10^−2^	<10^−6^	ns	ns
Total UFAs	<10^−6^	3.22 × 10^−2^	ns	7.80 × 10^−5^	ns	ns
Total SFAs	<10^−6^	3.22 × 10^−2^	ns	7.80 × 10^−5^	ns	ns
IV	<10^−6^	3.04 × 10^−6^	ns	<10^−6^	3.03 × 10^−3^	ns

*The significance of the R × D, R × T, and D × T first-order interaction effects are also provided*.

Similarly, model 1 ANOVA analysis also demonstrated highly significant ‘Between-Restaurant' differences between all aldehydic LOP analytes, with the exception of (Z*,E*)-alka-2,4-dienals (Table 3B). Indeed, this is a reflection of the significantly higher levels of these aldehydes found in Restaurant Y FF oil samples, although this observation is not simply consistent with considerations of the lower oxidative susceptibilities of samples analyzed than those of restaurant X, which is largely attributable to its higher [MUFA]:[PUFA] content ratio (IV values were *ca*. 8% higher for restaurant X FF oil samples, Figure 5). However, many further factors such as oil frying periods, frying conditions, oil reuse and frying temperatures, conjugated hydroperoxydiene degradation rate and extent, etc. may also exert major influences on these aldehyde concentrations. Moreover, Figure 7B shows that FF samples collected from restaurant Y had significantly greater oil contents than those from restaurant X. As observed for the acylglycerol fatty acid contents of these oils, there were significant differences “Between-Sampling Days-Within-Restaurants” for all of these aldehyde levels, bar 4-oxo-alkanals, and also that “Between-Sampling-Time-Points-within-Sampling Days” for two of the major α,β-unsaturated analytes. Notwithstanding, the Restaurant × Sampling Day interaction effect was significant for five of these aldehydes, and this provides evidence for the patterns of differences between these analyte responses varying significantly between sampling days, as noted above. Finally, the Restaurant × Sampling Time-Points interaction effect was found to be significant for 4-hydroxy-(*E*)-2-alkenals only, and the Sampling Days × Sampling Time-Points variance contribution was not significant for any of the analytes evaluated, as noted for the acylglycerol fatty acid analyses.

**Table 3B:** Statistical significance of “Between-Restaurants” (R), “Between-Sampling Days within Restaurants” (D), and “Between-Sampling Time-Points within Sampling Days” (T) sources of variation for seven aldehydic LOPs according to the Model 1 ANOVA-based hierarchal experimental design.

	**Restaurants (R)**	**Days (D)**	**Time-points (T)**	**R × D**	**R × T**	**D × T**
(*E*)-2-Alkenals	<10^−6^	3.13 × 10^−4^	2.81 × 10^−2^	5.21 × 10^−3^	ns	ns
(*E,E*)-Alka-2,4-dienals	<10^−6^	6.84 × 10^−4^	6.57 × 10^−2^	1.90 × 10^−4^	ns	ns
4,5-Epoxy-(*E*)-2-alkenals	<10^−6^	4.28 × 10^−2^	ns	ns	ns	ns
4-Hydroxy-(*E*)-2-alkenals	<10^−6^	<10^−6^	ns	ns	1.87 × 10^−2^	ns
(Z*,E*)-Alka-2,4-dienals	ns	5.23 × 10^−4^	ns	7.82 × 10^−3^	ns	ns
*n*-Alkanals	<10^−6^	5.02 × 10^−3^	ns	1.97 × 10^−4^	ns	ns
4-Oxo-alkanals	<10^−6^	ns	ns	3.66 × 10^−2^	ns	ns

*The significance of the R × D, R × T, and D × T first-order interaction effects are also provided*.

**Figure 9:**
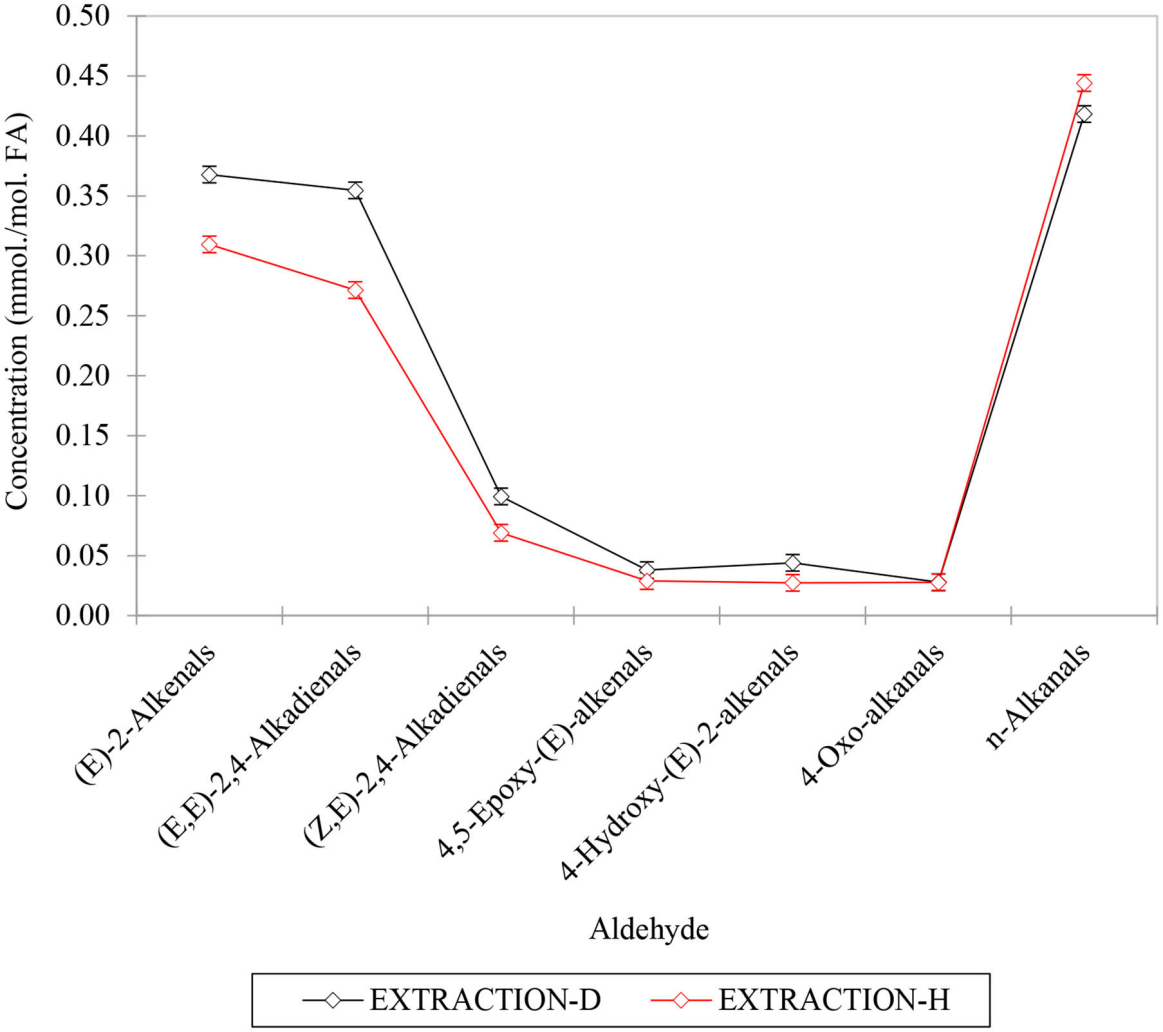
Plot of mean aldehyde concentrations determined using the direct C^2^HCl_3_ and exhaustive hexane extraction techniques (designated as EXTRACTION-D and –H, respectively). The differential responses of aldehydes to these extractions are responsible for the highly significant first-order aldehyde x extraction technique source of variation observed for the model 2 ANOVA performed.

**Figure 10:**
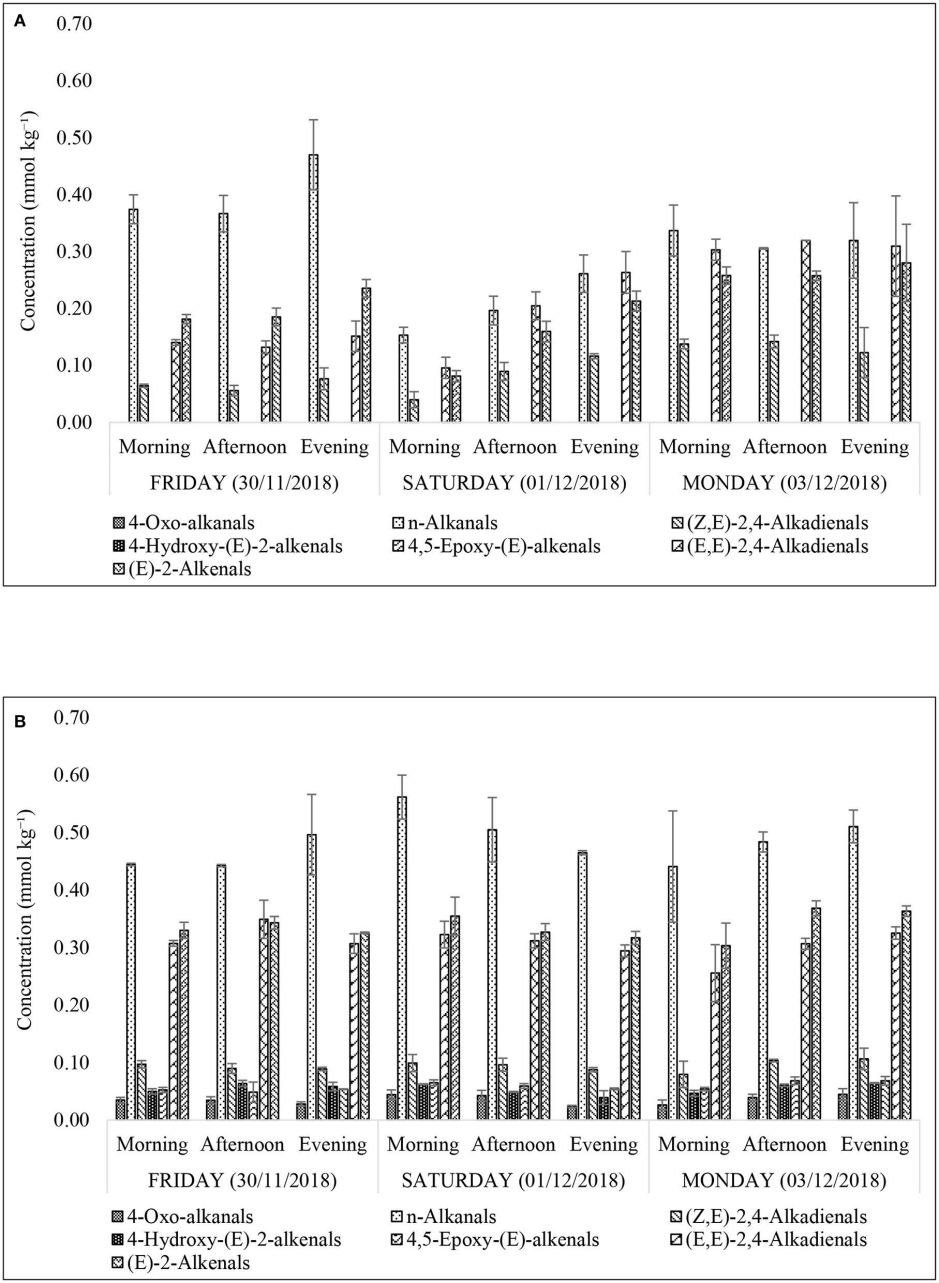
Concentrations of secondary aldehydic LOPs in culinary frying oil media extracted from FF samples purchased from restaurants X **(A)** and Y **(B)**. All values are presented as mean ± SD values.

For model 2, factorial ANOVA revealed that, in addition to the above expected significant differences between aldehyde classes analyzed and restaurants (*p* < 10^−6^ in each case), there was also a highly significant one between the extraction methods employed, with the C^2^HCl_3_ extraction process yielding overall mean *ca*. 15% increases in aldehyde concentrations determined for these analytes (*p* < 10^−6^). As noted above, this observation is conceivably attributable to small losses of selected aldehydes during the evaporation process employed for the *n*-hexane extractions. However, this was certainly not the case for all aldehydes detectable. Indeed, the Extraction Method × Aldehyde interaction source of variation was also statistically significant (Figure 10, *p* < 10^−6^), and this is best rationalized by the differences in extraction technique employed exerting a somewhat greater effect for some aldehyde classes rather than others; notably, ‘between-extraction methods' differences observed were statistically significant for all aldehyde analytes determined, except 4,5-epoxy-(E)-2-alkenals and 4-oxoalkanals. Nevertheless, Figure 9 shows that although the C^2^HCl_3_ extraction yielded higher aldehyde concentrations for (*E*)-2-alkenalS, (*E,E*)- and (*E,Z)*-2,4-alkadienals, all other aldehyde levels gave similar results for both extraction strategies (*n*-alkanals levels were, on average, 6% higher for the *n*-hexane extraction process).

Similarly, the Restaurant × Aldehyde interaction effect was also very highly statistically significant, but this is explicable by the differential patterns of aldehyde analytes detectable in extracts derived from FF samples collected from Restaurants X and Y, as noted above. Such differences are ascribable to the differing oils/oil blends employed by these vendors, with higher levels of (*E,E*)- and (*E,Z)*-2,4-alkadienals only arising from PUFA and not MUFA hydroperoxide sources. As expected, the Restaurant × Extraction Method source of variation was not found to be statistically significant.

### ICP-OES Determinations of the Trace Metal Compositions of FF Oil Extracts

Mineral elements in foods serve a healthy purpose in human nutrition. However, the value of mineral elements in culinary oils to human nutrition is minimal, and hence their inspection is geared toward the toxic effects that they may exert on healthy human cells (23). Table 4 highlights important trace metals detected in oil extracts in XFF and YFF, respectively. These trace metals may likely have been originally present in the culinary oils (i.e., from the natural source of the oil) used to fry the FFs and/or from the fry pot used in those commercial fast-food restaurants. Amongst the elements quantified in the oils extracted from the studied FF, Al, Ba, Fe, Pb, Ti, and Zn were present in considerable amounts in the FFs. Accordingly, the order of abundance of the metals in XFF oil was Al > Ba > Fe > Zn > Ti > Pb > Cu > Mn > Cr > Cd > Ni > V. That determined in YFF was in the order; Al > Ba > Fe > Zn > Ti > Pb > Cu > Cr > Cd > Mn > Ni > V (Table 4).

**Table 4:** ICP-OES profile of trace metals (μg) quantified in culinary oils (1.00 kg quantities) extracted from FF samples purchased from restaurants X and Y.

	**Trace metals**											
**Duration**	**Al**	**Ba**	**Cd**	**Cr**	**Cu**	**Fe**	**Mn**	**Ni**	**Pb**	**Ti**	**V**	**Zn**
**Restaurant X French fries (XFF)**												
Friday Morning	16414.17 ± 714.68	1761.28 ± 118.53	19.24 ± 3.11	33.68 ± 2.10	50.24 ± 5.13	822.66 ± 25.42	37.26 ± 2.24	< LOQ	159.25 ± 17.25	178.03 ± 2.78	4.79 ± 0.81	544.84 ± 27.17
Friday Afternoon	16692.42 ± 612.28	2228.16 ± 314.57	17.07 ± 4.09	24.47 ± 4.54	60.27 ± 6.50	2207.53 ± 60.26	48.61 ± 1.07	3.48 ± 0.26	165.62 ± 10.72	178.56 ± 8.72	5.10 ± 0.30	506.60 ± 46.42
Friday Evening	15192.66 ± 57.47	1590.48 ± 4.47	25.06 ± 4.38	33.27 ± 5.47	48.47 ± 15.12	1504.87 ± 379.62	39.45 ± 1.39	0.63 ± 0.02	178.69 ± 11.43	168.20 ± 4.57	1.67 ± 0.38	394.05 ± 26.93
**Daily Average**	**16099.75**	**1859.97**	**20.46**	**30.47**	**52.99**	**1511.69**	**41.77**	**2.05**	**167.86**	**174.93**	**3.85**	**481.83**
Saturday Morning	15543.90 ± 582.58	1630.45 ± 202.94	15.29 ± 4.99	27.27 ± 1.17	41.82 ± 6.16	671.25 ± 55.86	32.90 ± 3.64	< LOQ	355.10 ± 16.47	198.81 ± 7.99	2.08 ± 0.14	346.03 ± 3.61
Saturday Afternoon	14862.45 ± 500.74	1511.86 ± 31.04	23.25 ± 5.85	24.31 ± 6.47	40.28 ± 2.21	607.89 ± 15.96	29.86 ± 0.82	12.43 ± 1.41	137.64 ± 4.21	172.02 ± 12.07	3.73 ± 0.13	157.04 ± 8.24
Saturday Evening	14496.40 ± 597.93	1555.40 ± 141.82	7.08 ± 4.02	23.29 ± 1.04	33.84 ± 0.30	588.69 ± 73.61	28.76 ± 1.00	13.36 ± 3.93	137.31 ± 2.02	169.08 ± 6.81	1.50 ± 0.28	167.39 ± 17.61
**Daily Average**	**14967.58**	**1565.90**	**15.20**	**24.96**	**38.65**	**622.61**	**30.51**	**12.90**	**210.02**	**179.97**	**2.44**	**223.49**
Monday Morning	13476.22 ± 234.50	1338.83 ± 51.11	16.40 ± 2.59	34.44 ± 3.71	43.22 ± 5.80	670.96 ± 35.03	28.52 ± 2.11	27.81 ± 7.56	251.08 ± 27.49	159.17 ± 6.54	0.84 ± 0.16	196.20 ± 5.15
Monday Afternoon	13430.32 ± 442.80	1558.36 ± 232.10	10.78 ± 2.06	32.34 ± 4.19	41.42 ± 7.58	704.78 ± 43.34	31.82 ± 2.46	< LOQ	115.69 ± 10.35	178.97 ± 14.25	3.25 ± 0.22	320.03 ± 44.15
Monday Evening	3335.73 ± 645.02	1605.34 ± 273.78	21.07 ± 4.12	25.28 ± 6.67	28.15 ± 4.51	446.23 ± 35.10	13.40 ± 2.47	< LOQ	77.46 ± 7.29	186.24 ± 25.78	< LOQ	256.37 ± 17.96
**Daily Average**	**10080.76**	**1500.84**	**16.08**	**30.69**	**37.59**	**607.32**	**24.58**	**27.81**	**148.07**	**174.79**	**2.04**	**257.54**
**Overall Average**	**13716.03**	**1642.24**	**17.25**	**28.70**	**43.08**	**913.87**	**32.29**	**14.25**	**175.32**	**176.56**	**2.78**	**320.95**
^*^Daily maximum legal limit of trace metals (μg) per average body weight (77.5 kg)	11100^EFSA^	15500^EFSA^	190^EFSA^	23250^EFSA^	1100^PRI^	9000^FNB^	5500^EFSA^	217^EFSA^	1940^EFSA^	-	1800^FNB^	8250^PRI^
**Restaurant Y French fries (YFF)**												
Friday Morning	3939.23 ± 8.54	1918.34 ± 21.10	19.02 ± 1.60	34.04 ± 0.59	40.64 ± 3.05	620.84 ± 7.99	19.08 ± 0.36	< LOQ	102.70 ± 6.65	216.26 ± 8.91	< LOQ	628.46 ± 11.06
Friday Afternoon	3322.81 ± 261.73	1597.29 ± 118.27	14.91 ± 3.53	33.24 ± 1.43	28.25 ± 7.99	476.36 ± 61.51	13.18 ± 2.36	< LOQ	78.05 ± 2.40	182.26 ± 20.97	< LOQ	167.84 ± 18.55
Friday Evening	3348.56 ± 5.04	1519.65 ± 1.67	14.18 ± 0.95	19.68 ± 3.22	32.74 ± 1.30	457.52 ± 81.42	12.95 ± 0.63	< LOQ	76.48 ± 7.01	175.58 ± 10.30	< LOQ	253.04 ± 66.86
**Daily Average**	**3536.87**	**1678.43**	**16.04**	**28.98**	**33.88**	**518.24**	**15.07**	** < LOQ**	**85.74**	**191.37**	** < LOQ**	**349.78**
Saturday Morning	3593.10 ± 374.14	1628.71 ± 226.74	16.11 ± 3.33	23.50 ± 2.25	30.82 ± 3.20	370.98 ± 49.95	11.63 ± 1.38	< LOQ	80.77 ± 6.56	174.55 ± 12.95	< LOQ	403.34 ± 38.44
Saturday Afternoon	3296.38 ± 74.78	1523.34 ± 44.53	15.43 ± 2.09	20.94 ± 2.47	27.92 ± 7.16	319.64 ± 20.80	10.95 ± 0.11	< LOQ	58.87 ± 2.35	168.44 ± 0.69	< LOQ	198.47 ± 18.78
Saturday Evening	3228.98 ± 88.98	1469.79 ± 30.79	19.96 ± 3.96	33.06 ± 2.60	32.99 ± 0.78	427.81 ± 23.01	12.34 ± 0.07	< LOQ	59.57 ± 6.29	175.72 ± 4.00	< LOQ	184.94 ± 32.12
**Daily Average**	**3372.82**	**1540.61**	**17.17**	**25.83**	**30.58**	**372.81**	**11.64**	** < LOQ**	**66.40**	**172.90**	** < LOQ**	**262.25**
Monday Morning	3597.62 ± 289.23	1570.12 ± 185.24	19.59 ± 3.45	25.79 ± 4.88	37.95 ± 4.33	438.68 ± 60.79	13.26 ± 3.57	< LOQ	136.41 ± 23.96	194.01 ± 33.69	< LOQ	474.07 ± 32.32
Monday Afternoon	3377.66 ± 49.98	1492.37 ± 0.94	29.30 ± 2.91	24.15 ± 5.10	36.50 ± 6.75	366.52 ± 43.61	9.02 ± 0.62	< LOQ	63.51 ± 13.95	178.60 ± 17.72	< LOQ	386.75 ± 37.88
Monday Evening	3562.72 ± 246.27	1553.71 ± 89.71	14.32 ± 4.89	25.30 ± 3.97	30.88 ± 2.85	303.51 ± 61.54	8.28 ± 0.57	9.48	34.71 ± 5.38	172.78 ± 2.13	< LOQ	215.24 ± 29.05
**Daily Average**	**3512.67**	**1538.73**	**21.07**	**25.08**	**35.11**	**369.57**	**10.19**	**9.48**	**78.21**	**181.80**	** < LOQ**	**358.69**
**Overall Average**	**3474.12**	**1585.92**	**18.09**	**26.63**	**33.19**	**420.21**	**12.30**	**9.48**	**76.79**	**182.02**	** < LOQ**	**323.57**
^*^Daily maximum legal limit of trace metals (μg) per average body weight (77.5 kg)	11100^EFSA^	15500^EFSA^	190^EFSA^	23250^EFSA^	1100^PRI^	9000^FNB^	5500^EFSA^	217^EFSA^	1940^EFSA^	-	1800^FNB^	8250^PRI^

*Aluminum (Al), Barium (Ba), Cadmium (Cd), Chromium (Cr), Copper (Cu), Iron (Fe), Manganese (Mn), Nickel (Ni), Lead (Pb), Titanium (Ti), Vanadium (V) and Zinc (Zn). Less than limit of quantification (< LOQ). European Population Reference Intake (PRI), Food and Nutrition Board (FNB), European Food Safety Authority (EFSA)*,

* *Values are derived daily values. All values are presented as mean ± SD values. The bold values represent the mean daily average of values recorded in the morning, afternoon and evening*.

Overall, XFF oil extracts contained 3–5-fold Al, 2–3-fold Fe, and Mn, and 2-fold Ni and Pb concentrations than those found in the YFF oil extracts (Table 4). Al, by implication, may play an important role in Alzheimer's disease (24). This calls for scientific examination in regulating Al thresholds in foods. On the contrary, the concentrations of Ba, Cd, Cu, Cr, Cu, Ti, and Zn were comparatively similar for both XFF and YFF oil extracts (Table 4). It should also be noted that V was below the limit of quantification in all YFF oil extracts. However, although sparingly present, an overall average concentration of 2.78 μg V in 1.00 kg oil extract was measured in XFF oil. Likewise, an overall concentration of 9.48 and 14.25 μg Ni in 1 kg oil was measured in YFF and XFF oil extracts, respectively (Table 4).

Primarily, univariate two-sample *t*-tests, along with multivariate partial least squares-discriminatory analysis (PLS-DA) was conducted in order to explore “between-restaurant” differences between the levels and patterns of trace elements in extracted oil media isolated from FF samples. False discovery rate (FDR)-corrected *p-*values from *t*-tests performed were 2.29 × 10^−11^ for Al; 4.87 × 10^−10^ for Mn; 8.74 × 10^−8^ for V; 1,60 × 10^−5^ for Pb; 3.03 × 10^−5^ for Fe; 1,00 × 10^−2^ for Cu; and 1.63 × 10^−2^ Ni, which were all present at higher levels in the XFF product oils. All other elemental metals assessed in this manner were not statistically significant, although only Ti and Cd contents were greater in the YFF product oil matrix.

PLS-DA performed on the complete trace metal dataset revealed a powerful distinction between FF samples collected from the two restaurants, with only two samples from restaurant X being incorrectly classified (Figure 10). The highest Q^2^ cross-validation (CV) values obtained (via a 10-fold CV strategy) were those for models with 2 or 3 orthogonal components (Q^2^ = 0.69–0.71, a value of 0.50 serving as a threshold cut-off value for good agreement between predicted and observed classifications), and restaurant identity-based permutation testing *p*-values for these were < 5.0 × 10^−4^ (2,000 permutations). Variable importance parameters (VIPs) were 1.64 (Al), 1.58 (Mn), 1.45 (V). 1.27 (Pb), 1.23 (Fe), 0.85 (Cu), and <0.80 for all other trace elements. Similarly, a random forest (RF) computational intelligence analysis (CIA) model with 5 branches at each node and 1,000 trees correctly classified all restaurant Y samples (18/18) and 16/18 restaurant X ones, and hence the overall classification success rate was excellent (94.4%). See Supplementary Figure 13 for Three-dimensional PLS-DA plot.

Moreover, a principal component regression (PCR) model was developed in an attempt to relate aldehydic LOP levels to those of trace elements in the FF oil samples. However, with the exception of Ti content for 4-Hydroxy-(*E*)-2-alkenals, all trace metals detectable in the extracted oil samples negatively impacted on all aldehyde concentration values, i.e., their higher oil contents give rise to lower aldehydic LOP concentrations (Table 5).

**Table 5:** Statistically significant and non-significant FF oil sample trace metal variables and their contributions toward aldehydic LOP response variables in a PCR model (✓ indicates statistical significance; – and + signs in brackets represent the sign of correlations between these predictor and response variables, and where significant, *p*-values are also provided).

**Trace metals**	**(*E*)-2-Alkenals**	**(*E,E*)-2,4-Alkadienals**	**4,5-Epoxy-(*E*)-alkenals**	**4-Hydroxy-(*E*)-2-alkenals**	**(*Z,E*)-2,4-Alkadienals**	***n*-Alkanals**	**4-Oxo-alkanals**
Al	✓(–/*p* < 10^−6^)	✓(–/*p* = 6.06 × 10^−6^)	✓(–/*p* < 10^−6^)	✓(–/*p* < 10^−6^)	ns	✓(–/*p* = 5.96 × 10^−4^)	✓(–/*p* < 10^−6^)
Ba	ns	✓(–/ *p* = 1.15 × 10^−2^)	ns	ns	✓(–/*p* = 5.77 × 10^−3^)	ns	ns
Cd	ns	ns	ns	ns	ns	ns	ns
Cr	ns	ns	ns	ns	ns	ns	ns
Cu	✓(–/*p* = 1.85 × 10^−−2^)	✓(–/*p* = 5.51 × 10^−5^)	✓(–/*p* = 1.06 × 10^−3^)	✓(–/*p* = 7.97 × 10^−4^)	✓(–/*p* = 3.62 × 10^−2^)	ns	✓(–/*p* = 2.70 × 10^−3^)
Fe	✓(–/*p* = 1.16 × 10^−2^)	✓(–/*p* = 5.39 × 10^−4^)	✓(–/*p* = 2.31 × 10^−2^)	✓(–/*p* = 2.51 × 10^−2^)	✓(–/*p* = 1.64 × 10^−2^)	ns	✓(–/*p* = 4.25 × 10^−2^)
Mn	✓(–/*p* = 4.64 × 10^−6^)	✓(–/*p* = 1.48 × 10^−5^)	✓(–/*p* < 10^−6^)	✓(–/*p* < 10^−6^)	ns	✓(–/*p* = 1.14 × 10^−2^)	✓(–/*p* = 2.81 × 10^−6^)
Ni	ns	ns	✓(–/*p* 1.90 × 10^−2^)	✓(–/2.06 × 10^−2^)	ns	✓(–/*p* = 1.84 × 10^−2^)	✓(–/*p* = 3.57 × 10^−2^)
Pb	✓(–/*p* = 3.08 × 10^−3^)	ns	✓(–/*p* = 2.05 × 10^−3^)	✓(–/*p* = 5.93 × 10^−3^)	ns	✓(–/*p* = 7.04 × 10^−3^)	✓(–/*p* = 7.68 × 10^−3^)
Ti	ns	ns	ns	✓(+/*p* = 2.47 × 10^−2^)	ns	ns	ns
V	ns	ns	ns	ns	ns	ns	ns
Zn	ns	ns	ns	ns	✓(–/*p* = 2.97 × 10^−2^)	ns	ns
Significant PCR Components:	PC1 and PC2	PC1	PC1 and PC2	PC1 and PC2	PC1	PC1 and PC2	PC1 and PC2

*Significant PCs are also provided. All aldehyde concentrations were found to be predicable from PCR PC 1, and in 5 cases, PC2 as well. Metal content variables significantly loading on PC1 were Al (loading vector 0.83), Ba (0.63), Cu (0.88), Fe (0.76), Mn (0.90), Pb (0.67), and V (0.52); those significantly loading on PC2 were Ni (0.74), Ti (−0.73), and Zn (−0.65)*.

## Discussion

### Lipid Oxidation Products (LOPs)

The presence of α,β-unsaturated aldehydes (α,β-UA), namely (*E*)-2-alkenals; (*E,E*)-2,4-alkadienals; 4,5-epoxy-(*E*)-alkenals; 4-hydroxy-(*E*)-2-alkenals; and (*Z,E*)-2,4-alkadienals in the FF samples raises health concerns regarding the use of PUFA- and MUFA-containing culinary oils for high-temperature, peroxidation-promoting frying episodes. However, somewhat less toxic saturated aldehydes (*n*-alkanals) were found to be present at the highest concentrations in the XFF oil extracts (0.47 ± 0.06 mmol kg^−1^, Figure 10A), and also in YFF oil (0.56 ± 0.04 mmol kg^−1^, Figure 10B). An important observation was that the thermo-oxidized culinary oil used by XFF restaurant was changed between Friday evening and Saturday morning as evidently shown in Figures 4A, 5, 7A, 10A. Interestingly, *n*-alkanals, (*Z,E*)-2,4-alkadienals, (*E,E*)-2,4-alkadienals and (*E*)-alkenals all increase continuously throughout Saturday, and reach a maximum on Monday (Figure 10A). This suggests that the abovementioned LOPs concentrations first increase after replacing the culinary oil, but are then consumed by their reactions with potato biomolecules, perhaps free amino acids and protein lysine residues with reactive side-chain-NH_2_ functions via Schiff base and Michael addition processes. Since α,β-unsaturated aldehydes are more reactive toward biomolecules than saturated *n*-alkanals, this may explain why higher FF levels of (*Z,E*)-2,4-alkadienals; (*E,E*)-2,4-alkadienals; and (*E*)-alkenals were observed at the later sampling time-points. A similar observation was made in Moumtaz et al. (12). Restaurant Y showed consistent patterns of LOPs over the course of all 3 sampling days (Figure 10B). It is also worthy of note that the concentrations of all aldehydic LOPs in FF samples collected from restaurant Y are greater than those found for restaurant X. The reason may be that culinary oil used in frying YFF may have been subjected to a more prolonged frying duration than that used for the XFF, hence leading to a higher evolution of LOPs in YFF oil extracts.

Reference to current literature in order to determine the molecular nature of the acylglycerol identities of the culinary oils used for both restaurants was inconclusive, suggesting that those used for both restaurants were blends, potentially combinations of sunflower and rapeseed and/or soybean oils (25–31). In addition to these potential oil blends, the durations that FFs were exposed to the frying media may also influence the variations in aldehydic LOPs compositions, as noted above. The presence of aldehydic LOPs in FF oils demonstrates and provides evidence for the passive transfer of oxidation products from thermo-oxidized oil into fried foods. This, therefore, presents a major public health concern, since LOPs detectable in fried foods have been reported to be both cytotoxic and genotoxic. Indeed, they are strongly implicated in the pathogenesis of a wide range of chronic non-communicable human diseases (NCDs), for example their abilities to induce or propagate atherosclerosis, liver function impairments, teratogenicity, and gene mutation and carcinogenesis, as well as neurodegenerative diseases such as Huntington's, Alzheimer's, and Parkinson's disease (32).

It should also be noted that the ^1^H NMR signals of acrylamide, which is also a fertility and reportedly nervous system toxin (32), were undetectable in the generated spectra for XFF and YFF oils, but this is not unexpected since this analyte is very polar and has a very high solubility in water. Hence, if present in XFF and YFF samples, acrylamide would not be expected to passively transfer from the extracted FFs into the oil analysis medium.

Presently, a sizeable proportion of the human population opt to consume a variable range of fried foods on a weekly basis. Most importantly, highly heat-susceptible oxidation-prone unsaturated fatty acid-containing culinary oils are used as frying media at higher temperatures to generate fried foods. The general public has been oriented by the fact that unsaturated-rich cooking oils exert a favorable health impact on humans by elevating the blood levels of high-density-lipoprotein (HDL) cholesterol, and correspondingly suppressing those of low-density-lipoprotein (LDL) (33, 34). This is in major contrast to the adverse health effects associated with saturated fatty acid-rich culinary fats and oils, since they have been implicated in provoking or worsening cardiovascular and other chronic human diseases (35). Contrary to this narrative, saturated fatty acid-rich culinary fats and oils have a greater resistivity to the evolution of toxic LOPs than unsaturated fatty acid-laden culinary oils.

Furthermore, an *in vivo* study conducted in Madhya Pradesh, India showed that the blood lipid profile of 20 mice fed fast food-FFs, compared to a 20 mice control group fed a non-FF control diet, for 30 days, was analyzed to show an increment of 12.3% total cholesterol, 29.5% triacylglycerols, 25.8% low-density-lipoprotein (LDL), and 34.7% very-low-density-lipoprotein (VLDL). Simultaneously, high-density lipoprotein (HDL) levels in experimental mice blood lipid profiles decreased by 31.5% (36). This result provides an indication that continuous feeding of FFs could potentially increase the risk of obesity and cardiovascular diseases (36).

In another study conducted in Tehran, Iran, Esfarjani et al. (37) analyzed thermo-oxidized culinary oils arbitrary collected from 50 fast food restaurants in five districts classified either as high, moderate or low socio-economic districts. From this investigation, the authors concluded that most of the thermo-oxidized culinary oils were degraded and contained hazardous secondary oxidation products, which could be passively transferred into food matrices during frying processes, and therefore could pose a public health risk to consumers (37).

These have been some of the vital warnings from researchers to consumers and the food industry, whilst seeking scientific alternatives targeted on combating the evolution of these oxidation products in thermally-stressed culinary oils and fried foods. For example, the use of antioxidants could viably retard the oxidation process in culinary oils at typical frying temperatures. Another alternative could be the enactment of governmental legislations which dictate when exactly thermally-stressed culinary oils should be discarded after pre-defined periods of use as a frying media in restaurants. Regular exchange of thermo-oxidized culinary oils, as observed here in restaurant X, could therefore represent a step toward diminishing the levels of toxic LOPs present in fried foods.

### LOPs Contents of 30 g Servings of FFs

The value of 30 g represents the weight of FFs that was used for the *n*-hexane oil extraction conducted. This value is considered by us to be much lower than a “worst case scenario” since certain individuals are known to have much higher mean daily intakes of this popular food commodity, which in some cases may exceed 200 g. Estimated levels of LOPs (μmol) per 30 g serving of XFFs and YFFs are shown in Table 6. Whilst there are no reported regulatory limits for these LOPs, their contents expressed per 30 g serving of FFs may present a potential health risk to fast-food restaurant FF consumers. Comparatively, YFF-sourced oil extracts predominantly contained higher levels of LOPs than those obtained from restaurant XFF (Table 6). The variation in the pattern of LOPs over the course of 3 days does not vary significantly for restaurant Y, suggesting the establishment of an equilibrium mixture, with *n*-alkanals predominating. Moreover, *n*-Alkanals were the predominant aldehydic species detectable in restaurant XFF samples. After changing the culinary oil, which was notable on Friday evening and Saturday morning XFF sampling time-points, the concentrations of the major ^1^H NMR detected LOPs, i.e., *n*-alkanals; (*Z,E*)-2,4-alkadienals, (*E,E*)-2,4-alkadienals, and (*E*)-2-alkenals, fell drastically. Indeed, on the following Saturday morning, their levels were found to increase again, and then rose on Monday, with levels remaining similar at all sampling time. This suggests that (*Z,E*)-2,4-alkadienals; (*E,E*)-2,4-alkadienals and (*E*)-2-alkenals are consumed by reaction with potato chip biomolecules over course of the week. Since fundamentally the presence of alkylating agents, whether they be epoxides or aldehydes, is undesirable, the impact of changing the culinary oil, limited to 1–2 working days of thermo-oxidation at a typical frying temperature of 180°C, could represent a health-related development concerning the suppression of LOP generation in culinary frying oils (Table 6).

**Table 6:** Amounts of LOPs (μmol. per 30 g quantity) of FF servings per day from restaurants X and Y.

	**Aldehydic LOPs**						
**Duration**	**4-Oxo-alkanals**	**n-Alkanals**	**(Z,E)-2,4-Alkadienals**	**4-Hydroxy-(E)-2-alkenals**	**4,5-Epoxy-(E)-alkenals**	**(E,E)-2,4-Alkadienals**	**(E)-2-Alkenals**
**Restaurant X French fries (XFF)**							
Friday Morning	–	34.64	6.00	–	–	13.01	16.92
Friday Afternoon	–	38.57	5.83	–	–	13.89	19.49
Friday Evening	–	59.49	9.59	–	–	19.10	29.94
**Daily Average**	**–**	**44.23**	**7.14**	**–**	**–**	**15.33**	**22.12**
Saturday Morning	–	14.62	3.85	–	–	9.18	7.75
Saturday Afternoon	–	16.74	7.62	–	–	17.47	13.62
Saturday Evening	–	24.85	11.06	–	–	25.06	20.27
**Daily Average**	**–**	**18.74**	**7.51**	**–**	**–**	**17.24**	**13.88**
Monday Morning	–	38.10	15.52	–	–	33.99	28.90
Monday Afternoon	–	30.54	14.25	–	–	31.94	25.71
Monday Evening	–	34.35	13.06	–	–	33.13	30.05
**Daily Average**	**–**	**34.33**	**14.28**	**–**	**–**	**33.02**	**28.22**
**Overall Average**	**–**	**32.33**	**14.28**	**–**	**–**	**33.02**	**28.22**
**Restaurant Y French fries (YFF)**							
Friday Morning	5.05	64.68	14.09	7.29	7.61	44.74	47.98
Friday Afternoon	4.66	59.31	12.00	8.51	6.55	46.69	45.94
Friday Evening	4.21	74.51	13.31	8.76	8.03	45.85	48.72
**Daily Average**	**4.64**	**66.17**	**13.14**	**8.19**	**7.40**	**45.76**	**47.55**
Saturday Morning	6.74	85.64	15.10	9.54	9.92	49.19	54.10
Saturday Afternoon	6.87	81.93	15.75	8.00	9.64	50.73	53.19
Saturday Evening	3.78	74.18	13.97	6.28	8.68	46.89	50.50
**Daily Average**	**5.80**	**80.58**	**14.94**	**7.94**	**9.41**	**48.94**	**52.60**
Monday Morning	4.31	72.00	12.99	7.60	8.81	41.72	49.48
Monday Afternoon	6.77	83.94	17.96	10.51	11.85	53.10	63.82
Monday Evening	7.72	88.77	18.44	11.21	11.86	56.58	63.22
**Daily Average**	**6.27**	**81.57**	**16.46**	**9.77**	**10.84**	**50.46**	**58.84**
**Overall Average**	**5.57**	**76.11**	**14.85**	**8.63**	**9.22**	**48.39**	**52.99**

*The bold values represent the mean daily average of values recorded in the morning, afternoon and evening*.

In general, the concentration of *n*-alkanals in XFF oil extracts was 2–4-fold higher than that of YFF oil extracts (Table 6). Similarly, YFF oil extracts contained 2–3-fold greater (*E,E*)-2,4-alkadienal and (*E*)-2-alkenal levels than those in the XFF oil extracts. (*Z,E*)-2,4-Alkadienals in XFF oil extracts was at most 2-fold higher in YFF oil ones. Notwithstanding, appreciable amounts of 4-oxo-alkanals, 4-hydroxy-(*E*)-2-alkenals and 4,5-epoxy-(*E*)-alkenals, which were otherwise also present at trace amounts in XFF oil extracts, were quantifiable in the YFF oil extracts (Table 6). Overall, there was an increasing trend in the concentration of LOPs per sampling time per day, as expected in the case of continuous, repetitive heating episodes (i.e., frying reuse) throughout a long-time frame.

This investigation reports, for the first time, the “real-world” sampling of LOPs extracts from one of the most regularly consumed fried foodstuff in the world over a few days/times from two fast-food chain restaurants. French fries are prepared over a shorter time frame (between 2 and 5 min) in a continuously thermally-stressed culinary oil exposed to atmospheric O_2_, and predominantly at ≥180°C. Hence, the presence of significant amounts of LOPs in such a food renders it a potential public health hazard. This calls for important scientific measures to be tested and established to curtail LOPs evolution in thermo-oxidized oils, and therefore prevent their active transport into food matrices. The observation that changing the culinary oil during the large-scale cooking of FFs has only a short-term effect on LOPs concentrations is of considerable interest to those working in the food industry, and those focused on food safety regulations. Whilst the concentrations of LOPs observed may fall outside acceptable tolerances, it could be argued that regular changing of culinary oils employed for commercial frying practices is essential for limiting LOPs to their lowest possible level within the fast-food industry.

### Trace Metal Compositions

When compared with the daily maximum legal limit of trace metals (μg) per average body weight (77.5 kg) (Creff formula), Al and Fe (exclusive to Friday's daily average) determined in XFF oil extracts were inferred to be above the maximum legal limit proposed by European Population Reference Intake (PRI), Food and Nutrition Board (FNB) and European Food Safety Authority (EFSA) (Table 4). The other trace metals in XFF oil extracts were all below the maximum legal thresholds. Oil extracts of YFF contained trace metals whose levels were all below the maximum legal thresholds (Table 4). Nonetheless, this does not indicate that they cannot pose a health hazard to human consumers. Rather, all these trace metals, regardless of their concentrations, should be monitored and regulated in view of health concerns for consumers. The presence trace metal ions, both redox-active and non-redox-active ones in culinary oils, may be associated with autocatalysis of the oxidative degradation of the oils to LOPs, and passive transfer of the heavy metals and LOPs into fried foods will enhance toxicological concerns.

The observation that almost all trace metals detectable negatively impacted on frying oil sample aldehyde concentrations is, however, at variance with reports that both redox-active and non-redox-active metal ions can promote the peroxidation of PUFAs. Indeed, although the actions of Fe(II)/(III) and Cu(I)/(II) and other transition metal ions are readily explicable by their direct involvements in this process, for example both haem and non-haem iron ions in dietary peroxidation processes (38), non-redox-active metal ions such as Al(III) may facilitate the peroxidation of membrane lipids induced by iron ions (39). Indeed, in such reaction systems, it has been suggested that Al(III) ions may give rise to a complexation-dependent alteration in lipid membrane structure that stimulates lipid peroxidation (39). However, extrapolation of this postulate to predominantly triacylglycerol-containing culinary oil samples is not readily achievable, although in principle Al(III) ions may be complexed by hydroperoxide functions available in conjugated hydroperoxydiene species, or by any (unprotonated) carboxylate functions in frying period-dependent liberated free fatty acids. According to Emanuel et al. (40), electron transfer in the transition metal ion-promoted peroxidation of PUFAs is preceded by the formation of essential co-ordination complexes with hydroperoxide-OOH function oxygen donors, postulated to be with lipid hydroperoxide ligands in the case of the peroxidized culinary oil/FF samples investigated here. Notwithstanding, it has also been reported that in some cases, transition metal ions act as antioxidants, and such activities may arise from the direct neutralization of primary carbon-centered pentadienyl radicals, and/or peroxyl radicals generated therefrom (equations 2 and 3, respectively) (41).

(2) $$\begin{array}{l} {\text{R}}^{∙}+{\text{M}}^{\left(\text{n}+1\right)+}\;\to {\text{R}}^{+}+\text{M}{\text{e}}^{\text{n}+} \end{array}$$

(3) $$\begin{array}{l} \text{RO}{\text{O}}^{∙}+{\text{M}}^{\text{n}+}\text{ v RO}{\text{O}}^{-}+\text{M}{\text{e}}^{\left(\text{n}+1\right)+} \end{array}$$

Therefore, from results obtained in the current study, it certainly appears that the metals detectable in oil samples evaluated, and most especially elevated transition metal levels found in the XFF samples, may serve as anti- rather than pro-oxidants, and may offer this oil some level of protection against thermally-induced peroxidation.

However, (24) explored the influence of added metal ions on the generation of aldehydic peroxidation products in thermally-stressed canola oil, and found increases in the formation rates of such LOPs, particularly formaldehyde, acetaldehyde, propanal, and heptenal. Indeed, added Cu ions proved to be the most highly-rated catalyst in this context, followed by the effects exerted by added iron and aluminum ions.

Legislated metals such as As, Cr, Cd, and Pb are noted for their toxicity and metabolic roles in promoting the peroxidation of PUFAs in lipidic media, a process facilitating the generation of LOPs such as hydroperoxides, aldehydes, ketones, epoxides, and carboxylic acids (42, 43). Moreover, Trindade et al. (44) warned that Cu, Fe, Ni, and Zn detectable in culinary oils, may accelerate the oxidation of PUFAs, and hence give rise to the production of several types of oxidation products in the oils. If uncontrolled, such LOPs could potentially serve to amplify the adverse health effects putatively stemming from excessive fried food intake in humans (30).

### Trace Metal Contents of 30 g FF Servings

Comparison of the ICP-OES dataset with the LOPs and lipid acyl/free fatty acid profiles indicated that concentrations of linoleic and linolenic acyl groups were elevated for restaurant X (Figure 4A), which may be a reflection of the elevated Al and Fe ion concentrations (Table 4). However, overall, the concentration of LOPs does not simply correlate with these elevated Al and Fe concentrations (Table 4). Indeed, XFF oil (higher Al and Fe concentration) has lower concentrations of virtually all aldehydic LOPs (Figure 10A) when compared with the YFF oil (Figure 10B). This therefore suggests that another variable is limiting the impact that metal concentrations are having on this experiment. However, Fe levels are very small relative to the aldehyde ones, and therefore may not be expected to contribute much to this anyway (45).

Table 7 highlights the amounts of trace metals (μg) quantified in 30 g servings of XFF and YFF samples, respectively, which are compared to the daily maximum legal limit of trace metals (μg) per average body weight (77.5 kg) (46, 47). The results show that the studied trace metals quantified in oil extracts of XFF and YFF per 30 g serving were all below the maximum limit proposed by PRI, FNB and EFSA. Supplementary Table 7 describes the portions of FF (1 portion = 30 g FF) that may have to be consumed to meet the daily maximum legal limit of trace metals, taking into consideration an average body weight of 77.5 kg. For example, based on the 30 g per serving FF data presented on Table 5, 33.80–79.65 portions (1,014–2389.5 g) of XFF may have to be consumed to meet the daily maximum legal limit of Al (μg) per average body weight (77.5 kg) (Supplementary Table 7). Similarly, a 130.78–182.97 μg Al/kg oil extract from YFF (Table 7) would require a 77.5 kg average body weight person to consume 60.82–86.54 portions (1824.60–2596.2 g) of YFF to meet such a limit (Supplementary Table 7). The unavailability of data for the daily maximum legal limit of trace metals (μg) per average body weight (77.5 kg) for Ti renders it difficult to infer from the experimentally quantified Ti levels in XFF and YFF oil extracts.

**Table 7:** Contents of trace metals of FFs (μg per 30 g servings) from restaurant X and Y in comparison with their daily thresholds.

	**Trace metals**											
**Duration**	**Al**	**Ba**	**Cd**	**Cr**	**Cu**	**Fe**	**Mn**	**Ni**	**Pb**	**Ti**	**V**	**Zn**
**Restaurant X French fries (XFF)**												
Friday Morning	255.12	27.37	0.30	0.52	0.78	12.79	0.58	< LOQ	2.48	2.77	0.07	8.47
Friday Afternoon	336.58	44.93	0.34	0.49	1.22	44.51	0.98	0.07	3.34	3.60	0.10	10.22
Friday Evening	445.46	46.63	0.73	0.98	1.42	44.12	1.16	0.02	5.24	4.93	0.05	11.55
**Daily Average**	**345.72**	**39.65**	**0.46**	**0.66**	**1.14**	**33.81**	**0.91**	**0.04**	**3.68**	**3.77**	**0.08**	**10.08**
Saturday Morning	251.84	26.42	0.25	0.44	0.68	10.88	0.53	< LOQ	5.75	3.22	0.03	5.61
Saturday Afternoon	197.27	20.07	0.31	0.32	0.53	8.07	0.40	0.17	1.83	2.28	0.05	2.08
Saturday Evening	236.57	25.38	0.12	0.38	0.55	9.61	0.47	0.22	2.24	2.76	0.02	2.73
**Daily Average**	**228.56**	**23.96**	**0.22**	**0.38**	**0.59**	**9.52**	**0.47**	**0.19**	**3.27**	**2.75**	**0.04**	**3.47**
Monday Morning	306.17	30.42	0.37	0.78	0.98	15.24	0.65	0.63	5.70	3.62	0.02	4.46
Monday Afternoon	241.34	28.00	0.19	0.58	0.74	12.66	0.57	< LOQ	2.08	3.22	0.06	5.75
Monday Evening	70.83	34.09	0.45	0.54	0.60	9.48	0.28	< LOQ	1.64	3.95	< LOQ	5.44
**Daily Average**	**206.11**	**30.84**	**0.34**	**0.63**	**0.77**	**12.46**	**0.50**	**0.63**	**3.14**	**3.60**	**0.04**	**5.22**
**Overall Average**	**260.13**	**31.48**	**0.34**	**0.56**	**0.83**	**18.60**	**0.62**	**0.29**	**3.37**	**3.37**	**0.05**	**6.26**
*Daily maximum legal limit of trace metals (μg) per average body weight (77.5 kg)	11100^EFSA^	15500^EFSA^	190^EFSA^	23250^EFSA^	1100^PRI^	9000^FNB^	5500^EFSA^	217^EFSA^	1940^EFSA^	–	1800^FNB^	8250^PRI^
**Restaurant Y French fries (YFF)**												
Friday Morning	150.08	73.08	0.72	1.30	1.55	23.65	0.73	< LOQ	3.91	8.24	< LOQ	23.94
Friday Afternoon	107.41	51.63	0.48	1.07	0.91	15.40	0.43	< LOQ	2.52	5.89	< LOQ	5.43
Friday Evening	134.84	61.19	0.57	0.79	1.32	18.42	0.52	< LOQ	3.08	7.07	< LOQ	10.19
**Daily Average**	**130.78**	**61.97**	**0.59**	**1.05**	**1.26**	**19.16**	**0.56**	** < LOQ**	**3.17**	**7.07**	** < LOQ**	**13.19**
Saturday Morning	150.54	68.24	0.68	0.98	1.29	15.54	0.49	< LOQ	3.38	7.31	< LOQ	16.90
Saturday Afternoon	157.33	72.71	0.74	1.00	1.33	15.26	0.52	< LOQ	2.81	8.04	< LOQ	9.47
Saturday Evening	147.80	67.28	0.91	1.51	1.51	19.58	0.56	< LOQ	2.73	8.04	< LOQ	8.47
**Daily Average**	**151.89**	**69.41**	**0.78**	**1.17**	**1.38**	**16.79**	**0.52**	** < LOQ**	**2.97**	**7.80**	** < LOQ**	**11.61**
Monday Morning	171.97	75.05	0.94	1.23	1.81	20.97	0.63	< LOQ	6.52	9.27	< LOQ	22.66
Monday Afternoon	182.68	80.72	1.58	1.31	1.97	19.82	0.49	< LOQ	3.43	9.66	< LOQ	20.92
Monday Evening	194.26	84.72	0.78	1.38	1.68	16.55	0.45	0.52	1.89	9.42	< LOQ	11.74
**Daily Average**	**182.97**	**80.16**	**1.10**	**1.31**	**1.82**	**19.11**	**0.52**	**0.52**	**3.95**	**9.45**	** < LOQ**	**18.44**
**Overall Average**	**155.21**	**70.51**	**0.82**	**1.18**	**1.49**	**18.36**	**0.54**	**0.52**	**3.36**	**8.11**	** < LOQ**	**14.41**
*Daily maximum legal limit of trace metals (μg) per average body weight (77.5 kg)	11100^EFSA^	15500^EFSA^	190 ^EFSA^	23250^EFSA^	1100^PRI^	9000^FNB^	5500^EFSA^	217^EFSA^	1940^EFSA^	–	1800^FNB^	8250^PRI^

Aluminum (Al), Barium (Ba), Cadmium (Cd), Chromium (Cr), Copper (Cu), Iron (Fe), Manganese (Mn), Nickel (Ni), Lead (Pb), Titanium (Ti), Vanadium (V) and Zinc (Zn). Less than limit of quantification (LOQ). European Population Reference Intake (PRI), Food and Nutrition Board (FNB), European Food Safety Authority (EFSA), *Values are derived daily values.

Overall, the results of this study where samples were taken from chain restaurants, with standardized and enforced operating procedures, suggest that there could be considerable variation in LOPs and trace metals found in FFs being prepared by smaller independent restaurants, which the authors shall investigate further. This builds on the work already undertaken by the authors in this area, which was recently published (48).

## Conclusions

Over the course of 3 days and 3 different diurnal time periods, the present study established that between the two UK fast-food restaurants, the total lipid acylglycerol content extracted and established as a percentage of FF mass, was directly proportional to the levels of LOPs identified and quantified in these samples. Total lipid acylglycerol contents were consistently higher in YFF [13.2–17.3% (w/w)] than in XFF [8.2–11.9% (w/w)], and hence so were the identified and quantified aldehydic LOPs. Overall, with regards to the ^1^H NMR analyses of the major acyl function resonances, the classes of the fatty acid contents were quite distinct between XFF and YFF oil extracts. Moreover, small differences in the amounts of the fatty acid contents between XFF and YFF oils were observed. For example, the total ω-3 acylglycerols and linolenoylglycerol levels were both higher in the XFF than the YFF FF oils extracted. Although both primary and secondary LOPs identified in the oil extracts of FFs across the sampling time-points were similar, YFF oil extracts were found to be richer in all LOPs determined. In retrospect, the presence of LOPs in FFs studied present a health hazard to consumers regarding the use of UFA-rich culinary oils for high-temperature, peroxidation-promoting frying episodes. Moreover, levels of redox-active metals measured with the aid of ICP-OES in oil extracts of FFs were found to be inversely correlated with FF oil LOP concentrations, and this may indicate that the redox-active ones monitored (e.g., those of Fe, Cu, and Mn) may exert antioxidant rather than pro-oxidant roles.

## Data Availability Statement

The original contributions presented in the study are included in the article/Supplementary Material, further inquiries can be directed to the corresponding author/s.

## Author Contributions

All authors listed have made a substantial, direct and intellectual contribution to the work, and approved it for publication.

## Conflict of Interest

The authors declare that the research was conducted in the absence of any commercial or financial relationships that could be construed as a potential conflict of interest.
