# Supplementary material for: “Real-World” Evaluation of Lipid Oxidation Products and Trace Metals in French Fries From Two Chain Fast-Food Restaurants
Source: Front Nutr. 2021 Feb 5;8:620952. doi: 10.3389/fnut.2021.620952 (PMC7892784; doi:10.3389/fnut.2021.620952)
Supplement: Supplementary file 1 [file Data_Sheet_1.DOCX]

**‘Real-world’ evaluation of lipid oxidation products and trace metals in French fries from two chain fast-food restaurants**

**Adam LE GRESLEY^a*^, Gilbert AMPEM^a^, Simon DE MARS^a†^, Martin GROOTVELD^b^ and Declan P NAUGHTON^a^**

**^a^Department of Chemistry and Pharmaceutical Sciences, Kingston University, Kingston-upon-Thames, Surrey, KT1 2EE, UK.**

^b^Leicester School of Pharmacy, De Montfort University, Leicester, LE1 9BH, UK.

**† This paper is dedicated to Simon De MARS, a dear colleague who passed away suddenly in 2019. He was responsible for obtaining the ICP-OES data.**

**^*^Corresponding author:**

**Dr Adam Le Gresley, Tel + 44 (0)20 84177432 Email:** [a.legresley@kingston.ac.uk](mailto:a.legresley@kingston.ac.uk)

Supporting Information

SI.1 Methods

SI.1.1 Extraction of oil from French fries


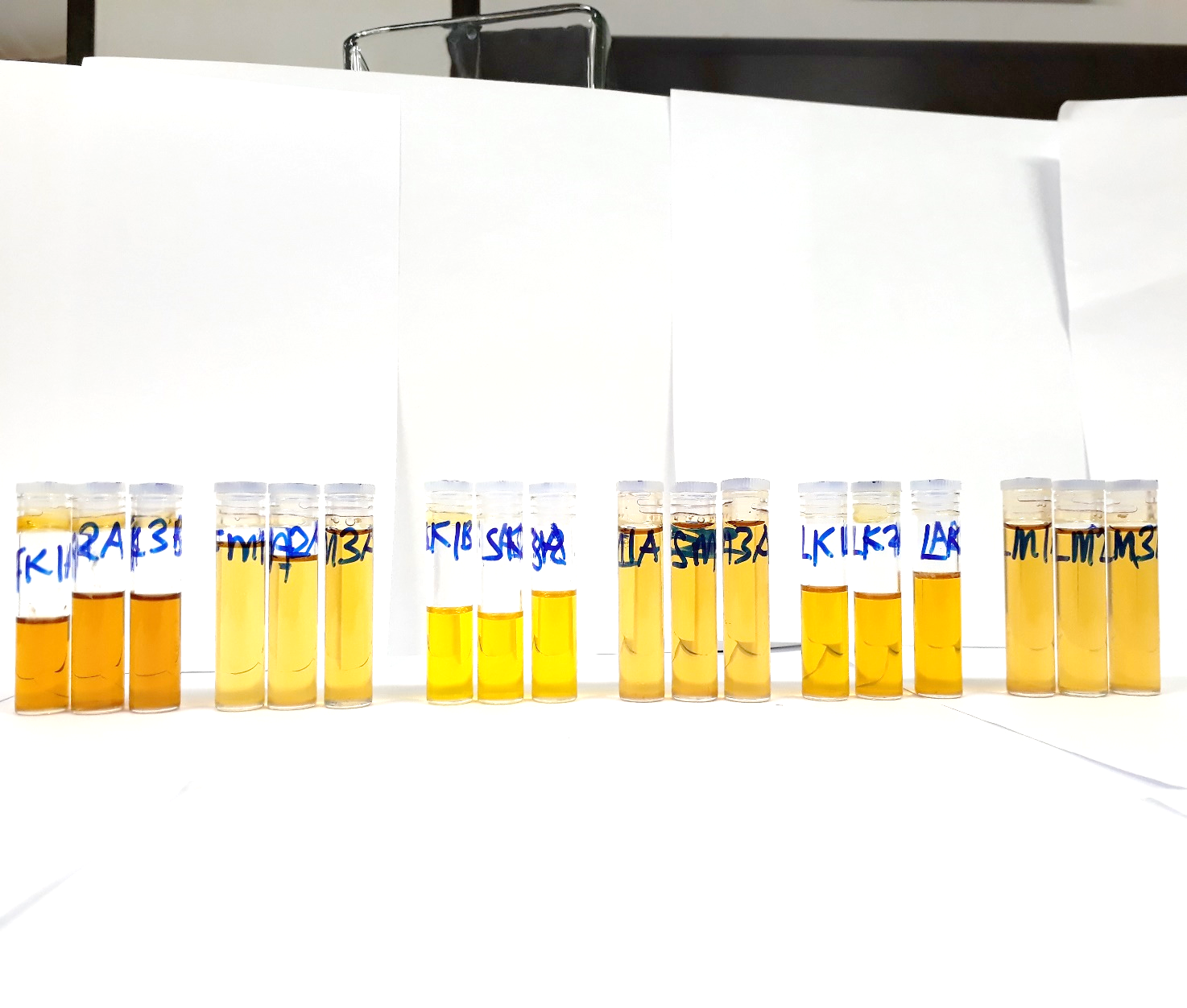


XFM

XFA

XFE

YFM

YFA

YFE

XSM

XSA

XSE

YSM

YSA

YSE

XMM

XMA

XME

YMM

YMA

YME

**Figure S.1.** Culinary oils extracted from FF samples purchased from restaurants X and Y. Abbreviations (from left to right): Restaurant X Friday Morning (XFM), Restaurant X Friday Afternoon (XFA), Restaurant X Friday Evening (XFE), Restaurant Y Friday Morning (YFM), Restaurant Y Friday Afternoon (YFA), Restaurant Y Friday Evening (YFE), Restaurant X Saturday Morning (XSM), Restaurant X Saturday Afternoon (XSA), Restaurant X Saturday Evening (XSE), Restaurant Y Saturday Morning (YSM), Restaurant Y Saturday Afternoon (YSA), Restaurant Y Saturday Evening (YSE), Restaurant X Monday Morning (XMM), Restaurant X Monday Afternoon (XMA), Restaurant X Monday Evening (XME), Restaurant Y Monday Morning (YMM), Restaurant Y Monday Afternoon (YMA), Restaurant Y Monday Evening (YME).

SI.1.2 Proton NMR measurements

The compositions of XFF and YFF oils were analysed on a Bruker Ultrashield 600 spectrometer (Kingston University, London) operating at 600.13 MHz frequency and a 298 K probe temperature. The acquisition parameters for the instrument were: size of fid 65,536; number of scans 256; probe temperature 300 K; spectral width 20.573 ppm; relaxation delay 1.000 s; acquisition time 4.819 s; pulse width 90°C; and total acquisition time 16 mins. (Le Gresley *et al*., 2019).

Aliquots of 0.30 mL of XFF and YFF oils were diluted with 0.60 mL deuterated chloroform (C^2^HCl_3_) (99.8% purity). From the 0.90 mL resulting lipid-C^2^HCl_3_ mixture, 0.50 mL was transferred to 5-mm diameter NMR tubes (Norrell HT, GPE Scientific) to which was added 0.10 mL of 1,3,5-tribromobenzene (TBB) solution (prepared by dissolving 4.14 mg TBB in 2.0 mL C^2^HCl_3_). In addition to acting as a solvent, C^2^HCl_3_ provided a field frequency lock for the oils. Also, TBB, having a δ-scale value of 7.537 ppm, served as an internal quantitative ^1^H NMR standard in the determination of NMR-detectable autoxidation products in the culinary oil extracts. The ^1^H δ-scale values of all signals in the profiles were referenced to tetramethylsilane (TMS) (δ = 0.000 ppm) and/or residual chloroform (δ = 7.283 ppm) (Le Gresley *et al*., 2019).

SI.1.3 Analysis of acyl groups

Acyl groups identified from the ^1^H NMR spectra of the oils were reported as molar percentages. This was based on the assumption that the areas of ^1^H resonances in spectra are proportional to the product of number of ^1^H nuclei generating them, and the concentration of the triacylglycerols present. Computations of the molar percentages of the acyl groups were performed in triplicate, and the results are provided as mean±SD values.

SI.1.4 Analysis of iodine values (IVs)

Iodine value (IV) was calculated based on the method and formula developed and validated by Guillén and Ruiz (2003). Iodine value was evaluated in triplicate, and the results are provided as mean±SD values.

SI.1.5 Analysis of lipid oxidation products

Identification of LOPs were primarily performed with the aid of literature ^r1^. Carbon-13 (^13^C) and ^13^C Distortionless Enhancement by Polarization Transfer (^13^C DEPT-90) spectra, together with other two-dimensional NMR spectroscopic techniques, i.e. Heteronuclear Single Quantum Correlation (HSQC) and Correlation Spectroscopies (COSY) (Le Gresley *et al*., 2019), were also employed for this purpose.

To distinguish the different types of ^13^C signals, ^13^C DEPT-90 NMR spectra were acquired and employed. Indeed, HSQC spectra showed the direct bond attachments existing between ^1^H and ^13^C in a compound, whereas that of ^1^H-^1^H COSY was useful in the determination of chemical groups underscoring pairs of ^1^H coupled to each other within a molecule (Le Gresley *et al*., 2019).

SI.1.6 Analysis of trace metals

**Table S.1** MARS microwave heating and cooling programme for the digestion of culinary oil samples

| Phase | Description | Time (min.) | Power (W) |
| --- | --- | --- | --- |
| 1 | Heating* | 2 | 400 |
| 2 | Cooling | 5 | - |
| 3 | Heating* | 2 | 800 |
| 4 | Cooling | 5 | - |
| 5 | Heating* | 2 | 800 |
| 6 | Cooling | 5 | - |
| 7 | Ventilation | 20 | - |

*Pressure (55 Bar) and temperature (240°C) were only applicable during the heating stage for the test samples.

SI.2 Derivations of key acyl group contents in culinary frying oils

Equations S1 to S8, which are based on those available in Martínez-Yusta *et al*. (2014), were employed to compute all oil contents of acylglycerol fatty acids.

Omega-3 acyl groups (%) = 100 × [*A*_B_ / (*A*_B_ + *A*_A_)] (SI1)

Oleic (or monounsaturated) acyl groups (%) = 100 × [(*A*_E_ – 2 *A*_G_ – *A*_H_) / 3 *A*_I_] (SI2)

Linoleic acyl groups (%) = 100 × (2 *A*_G_ / 3 *A*_I_) (SI3)

Linolenic acyl groups (%) = 100 × (2 *A*_H_ / 3 *A*_I_) (SI4)

Total Polyunsaturated acyl groups (%) = Linoleic (%) + Linolenic (%) (SI5)

Unsaturated acyl groups (%) = Oleic (%) + Linoleic (%) + Linolenic (%) (SI6)

Saturated and modified acyl groups (%) = 100 × [1 – (*A*_E_ / 3 *A*_I_)] (SI7)

Iodine value = 10.54 + 13.39 × [100 × (*A*_K_ / Total signal resonance intensities ranging from *A*_A_ to *A*_K_)] (SI8)

By definition:

*A*_A_ is the area of signal A (methylic protons of saturated, oleic and linoleic acyl groups)

*A*_B_ is the area of signal B (methylic protons of *ω*-3 acyl groups)

*A*_E_ is the area of signal E (*mono*-allylic protons of all unsaturated acyl groups)

*A*_G_ is the area of signal G (*bis*-allylic protons of linoleic acyl groups)

*A*_H_ is the area of signal H (*bis*-allylic protons of linolenic acyl groups)

*A*_I_ is the area of signal I (protons of carbon atoms 1 and 3 of the triacylglycerol glyceryl backbone)

*A*_K_ is the area of signal K (olefinic protons of all unsaturated acyl groups)

Equations SI1 to SI8 are based on the principle that the area of the signals generated from ^1^H NMR spectra are proportional to the number of protons that generate them.

SI.3 Characterisation of LOPs

SI.3.1 Primary LOPs

Primary LOPs are relatively short-lived intermediates that precede the formation of secondary LOPs. Resonances ascribable to primary LOPs are reportedly difficult to identify (Martínez-Yusta *et al*., 2014), despite ^1^H NMR analyses of these species being undertaken since the early 1990’s. Table S.2 provides a description of the primary LOPs profiled in Figure S.2, which were identified in both XFF and YFF oil extracts. The level of intensity observed for the signals found in each spectrum acquired is ascribable to the trend of oxidation observed for the acyl groups of XFF and YFF oil extracts. Oil extracts of YFF all had both (*E,E*)- and (*Z,E*)-conjugated hydroperoxydiene signals detectable. However, hydroperoxide groups were undetectable in XFF oil extracts (Figure S.2). Signal q is formic acid, which arises from the thermal degradation of malondialdehyde (MDA) (Le Gresley *et al*., 2019) (Figure S.2).


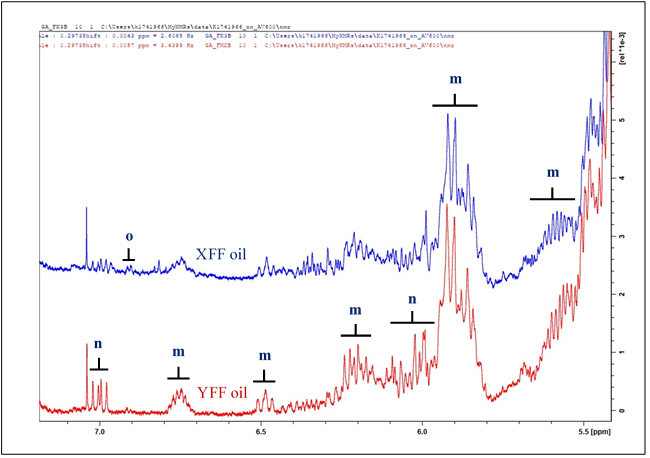

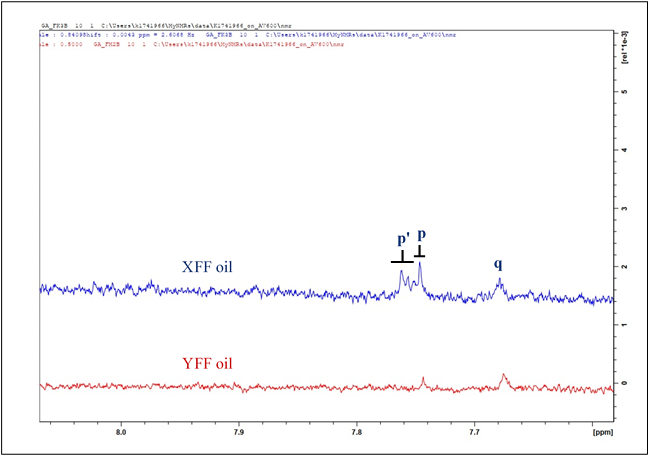

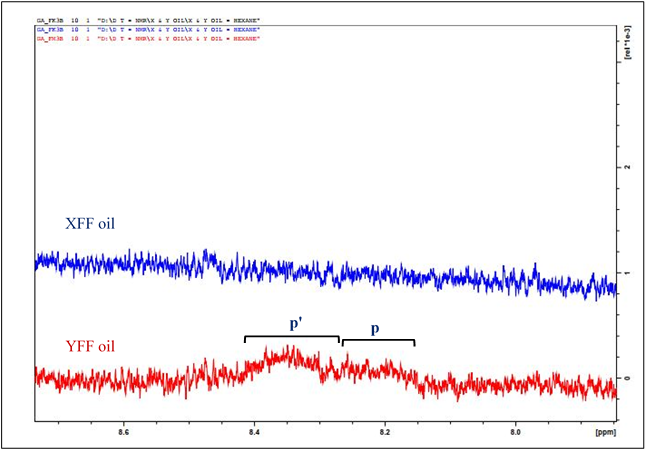


**Figure S.2.** ^1^H NMR spectra showing expanded 5.5–7.1 ppm regions of the ^1^H NMR profiles of XFF (blue) and YFF (red) oils (*n*-hexane-extracted), which reveal conjugated hydroperoxydienes and hydroxydienes (the former primary LOPs), and the olefinic resonances of α,β-unsaturated aldehydes. Broad resonances attributable to hydroperoxide groups (primary LOPs) present in the 8.0–8.6 ppm regions of culinary oils extracted from these FF samples are also shown. Signal p is a designated –OO**H** resonance of (*E*,*E*)- hydroperoxydienes and signal p' is a designated –OO**H** resonance (*Z,E*)-hydroperoxydienes. Abbreviations: Restaurant X french fries (XFF), Restaurant Y french fries (YFF). Letter assignments of resonances correspond to those provided in Table S.2.

**Table S.2** Assignment of bulk ^1^H NMR signals of conjugated hydroperoxydienes and hydroxydienes (the former a primary LOPs), and olefinic resonances of α,β-unsaturated aldehydes present in the ^1^H NMR profiles of XFF and YFF oils, including chemical shift values, multiplicities, and their associated functional group assignments.

|  |  |  | Functional group | |
| --- | --- | --- | --- | --- |
| Signal | Chemical shift (ppm) | Multiplicity | Condensed function | Classification |
| m | 5.526–5.624 | *ddm* | –C**H**═C**H**–C**H**═C**H**– | (*E*,*E*)-conjugated olefinic protons of CHPDs (the C2 vinylic proton of (*E*)-2-alkenals is at δ = 6.10 ppm, and the C3 one at δ = 6.85 ppm). Olefinic resonances of (*E*)-alka-2,4-dienals are located at 6.04, 6.20 and 6.30 ppm). Their C-3 olefinic proton has a multiplet signal located at δ = 7.07 ppm. |
| m | 5.822–5.952 | *ddm* | –C**H**═C**H**–C**H**═C**H**– |  |
| m | 6.139–6.253 | *ddd* | –C**H**═C**H**–C**H**═C**H**– |  |
| m | 6.448–6.513 | *ddtd* | –C**H**═C**H**–C**H**═C**H**– |  |
| m | 6. 699–6. 786 | *ddm* | –C**H**═C**H**–C**H**═C**H**– |  |
| n | 5.973–6.113 | *ddm* | –C**H**═C**H**–C**H**═C**H**– | (Z,*E*)-conjugated olefinic protons of CHPDs |
| n | 6.966–7.027 | *dddd* | –C**H**═C**H**–C**H**═C**H**– |  |
| o | 6.893–6.919 | *dd* | –C**H**═C**H**–C**H**═C**H**– | (Z,*E*)-conjugated olefinic protons of CHPDs |
| p/p' | 8.0–8.6 | - | –OO**H** | Hydroperoxide-OOH groups |
| q | 7.667–7.681 | *s* | **H**–CO_2_H | Formic acid |

Abbreviations: doublet (*d*), triplet (*t*), multiplet (*m*), doublet of doublets (*dd*), conjugated hydroperoxydienes (CHPDs). Letter assignments and characteristics correspond to those provided in Figures S.2 and S.9. The highlighted n signals contain significant contributions from the vinylic protons of (*E*)-2-alkenals (multiplets at δ = 6.12 and 6.86 ppm),

SI.3.2 Secondary LOPs

SI.3.2.1 Epoxides

Epoxides directly evolve from the oxidation of linoleic and oleic acyl groups (Goicoechea and Guillén, 2010; Guillén and Uriarte, 2012; Martínez-Yusta *et al*., 2014). Their presence in oil extracts of XFF and YFF samples also offer health hazards to consumers, and these includes carcinogenesis, cell death and organ malfunction (Markaverich *et al*., 2005; Thompson and Hammock, 2007). An inspection of each spectrum acquired revealed that signals ascribable to epoxides and primary alcohols identified in XFF and YFF oils were all similar in intensities across the sampling time-points (Figure S.3). An exception was the lower intensities of the signal t (9,10-Epoxy-octadecanoate; 9,10-Epoxy-12-octadecenoate (leukotoxin); and 12,13-Epoxy-9-octadecenoate (isoleukotoxin)), signal u (9,10–12,13-Diepoxyoctadecanoate), and signal v (primary alcohol LOP) resonances in XFF oil extracted from FFs purchased on a Saturday (01/12/2019). Similarly, a relatively lower intensity was also observed for signals q ((*E*)-9,10-Epoxystearate), r ((*Z*)-9,10-Epoxystearate), and x, the latter being an unidentified signal (Figure S.3).


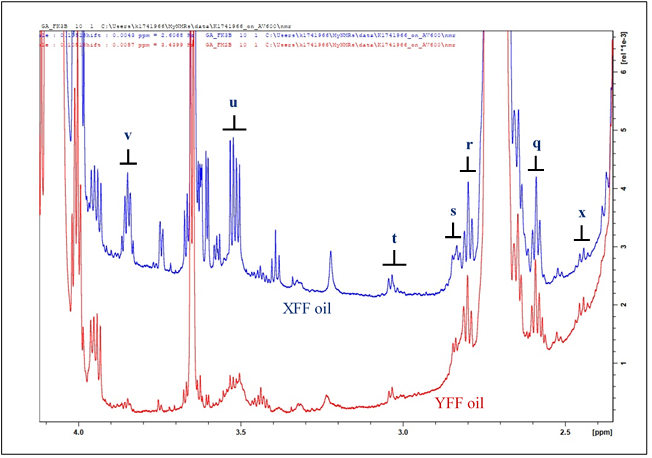


**Figure S.3.** Expanded 2.4–4.1 ppm regions of the ^1^H NMR spectra oils *n*-hexane-extracted from FF samples collected from restaurants X and Y, showing resonances ascribable to fatty acid epoxides (primary LOPs) and primary alcohols. Abbreviations: Restaurant X and Y French fries (XFF and YFF respectively). Letter assignments of resonances correspond to those provided in Table S.3.

**Table S.3** Assignment of bulk ^1^H NMR signals of fatty acid epoxides (primary LOPs) and primary alcohols present in the ^1^H NMR profiles of XFF and YFF oils, including chemical shift values, multiplicities, and their associated functional group assignments.

|  |  |  | Functional group | |
| --- | --- | --- | --- | --- |
| Signal | Chemical shift (ppm) | Multiplicity | Condensed function | Classification |
| x | 2.420–2.470 | *m* | - | Unidentified |
| q | 2.544–2.607 | *m* | –C**H**O**H**C– | (*E*)-9,10-Epoxystearate |
| r | 2.775–2.828 | *m* | –C**H**O**H**C– | (*Z*)-9,10-Epoxystearate |
| s | 2.828–2.854 | *m* | –C**H**OHC–CHO**H**C– | 9,10–12,13-Diepoxyoctadecanoate |
| t | 2.987–3.047 | *m* | –C**H**O**H**C– | 9,10-Epoxy-octadecanoate; 9,10-Epoxy-12-octadecenoate (leukotoxin); and 12,13-Epoxy-9-octadecenoate (isoleukotoxin) |
| u | 3.477–3.544 | *m* | –CHO**H**C–CH_2_–C**H**OHC– | 9,10–12,13-Diepoxyoctadecanoate |
| v | 3.822–3.868 | *m* | *α*-C**H**_2_ | Primary alcohol LOPs |

Abbreviations: multiplet (*m*). Letter assignments and characteristics correspond to those provided in Figures S.3 and S.10.

SI.3.2.2 Aldehydes

The aldehydic LOPs identified in XFF and YFF oils were (*E*)-2-alkenals, (*E,E*)-2,4-alkadienals, 4,5-epoxy-(*E*)-alkenals, 4-hydroxy-(*E*)-2-alkenals and (*Z,E*)-2,4-alkadienals (α,β-unsaturated aldehydes (*α*,β-UAs)), as well as *n*-alkanals and 4-oxo-alkanals (saturated aldehydes): Figure S.4 and Table S.4. Between the two restaurants, YFF oil extracts predominantly showed higher contents of aldehydic LOPs than those of XFF samples (Figure S.4). Their passive transfer from thermo-oxidized oils into foods cooked therein underscores the hazardous impact that they may exert.

**
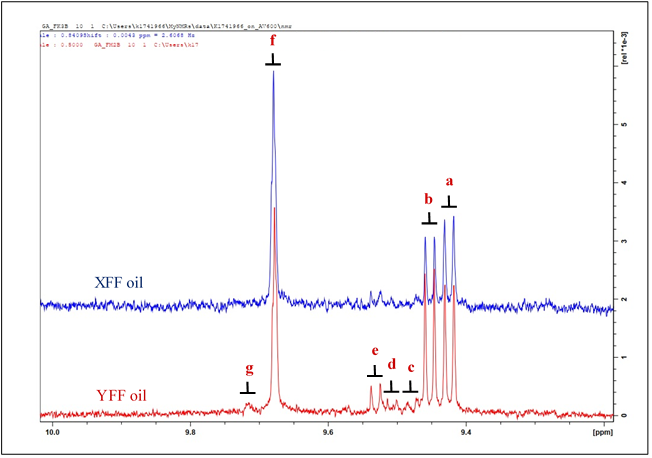
**

**Figure S.4.** ^1^H NMR spectra of frying oils collected from restaurants X and Y, showing aldehydes present within the 9.3–10.0 ppm regions of C^2^HCl_3_-reconstituted *n*-hexane-oil extracts of FF samples purchased from restaurants X and Y. Abbreviations: XFF and YFF, and Restaurant X and Y French fry samples respectively. Letter assignments of resonances correspond to those provided in Table S.4.

**Table S.4** Assignments of –C**H**O functional groups of ^1^H NMR signals of secondary aldehydic LOPs present in the ^1^H NMR profiles of XFF and YFF oils, including chemical shift values, multiplicities, and their associated functional groups.

|  |  |  | Functional group | |
| --- | --- | --- | --- | --- |
| Signal | Chemical shift (ppm) | Multiplicity | Condensed group | Classification |
| a | 9.408–9.437 | *d* | –C**H**O | (*E*)-2-Alkenals |
| b | 9.437–9.468 | *d* | –C**H**O | (*E*,*E*)-2,4-Alkadienals |
| c | 9.468–9.492 | *d* | –C**H**O | 4,5-Epoxy-(*E*)-alkenals |
| d | 9.492–9.514 | *d* | –C**H**O | 4-Hydroxy-(*E*)-2-alkenals |
| e | 9.514–9.542 | *d* | –C**H**O | (*Z*,*E*)-2,4-Alkadienals |
| f | 9.656–9.695 | *t* | –C**H**O | *n*-Alkanals |
| g | 9.706–9.721 | *t* | –C**H**O | 4-Oxo-alkanals |

Abbreviations: doublet (*d*), triplet (*t*). Letter assignments and characteristics correspond to those provided in Figures S.4 and S.11.

SI.4 Deuterated chloroform and *n*-hexane extraction techniques

SI.4.1 Major acyl groups and minor compounds

Deuterochloroform and *n*-hexane extraction techniques impacted on the intensities of the ^1^H NMR signals shown on Figure S.5 (i) [expanded regions of selected resonances are also shown in Figure S.5 (ii)]. In addition, minor compounds detectable are presented in Figure S.6. As expected, when the two extraction methods are directly compared, the *n*-hexane-oil extracts (hoe) of XFF and YFF showed greater intensities, and hence higher concentrations than those of their respective deuterated chloroform extracts (dce). This arises from the ^1^H NMR analysis of the intact extracted frying oil medium for the hexane extraction method, but not so for the deuterochloroform extracts, which involved a direct extraction of the FF samples consisting of only *ca.* 10-15% (w/w) oil content. Comparisons of the spectral signals of major acyl chains of Figure S.5, and minor compounds of Figure S.6, are consistent with the respective intensities of resonances in the spectra shown in Figures 3 and 6 (profiled for the oil extracts in the main manuscript). Expanded spectra of some selected major signals - specifically, signals A, B, C, E, G, H, J and K, showed that, with the exception of differences in intensities between-extraction methods and between-restaurants, qualitatively, all resonances present in the ^1^H NMR profiles were similar between restaurants and extraction techniques [Figure S.5 (ii)].

**(i)**


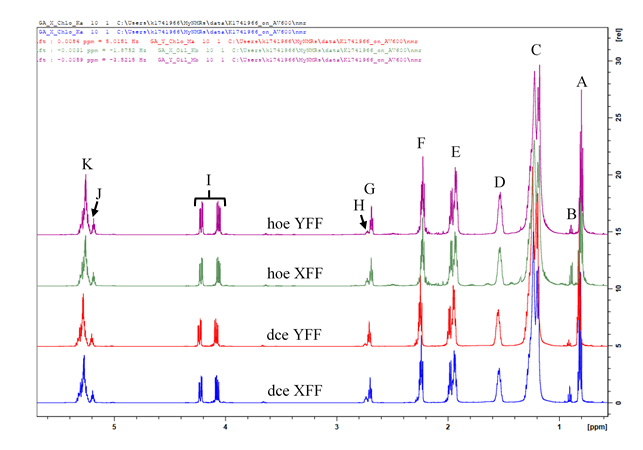


**Figure S.5.** ^1^H NMR spectra showing (i) major acylglycerol functions, and (ii) expanded regions of resonances A, B, C, E, G, H, J and K present within the 0.0–5.4 ppm regions of deuterochloroform and *n*-hexane-oil extracts of FF samples purchased from restaurants X and Y. Abbreviations: XFF and YFF, Restaurant X and Y French fries respectively; *Ln*, Linolenoyl signal; *L*, Linoleoyl signal; *O*, oleoyl signal; *S*, saturated fatty acid signal; dce, Deuterated chloroform extract; hoe, *n*-Hexane-oil extract. Letter assignments of resonances correspond to those provided in Table 1 described in the main manuscript.


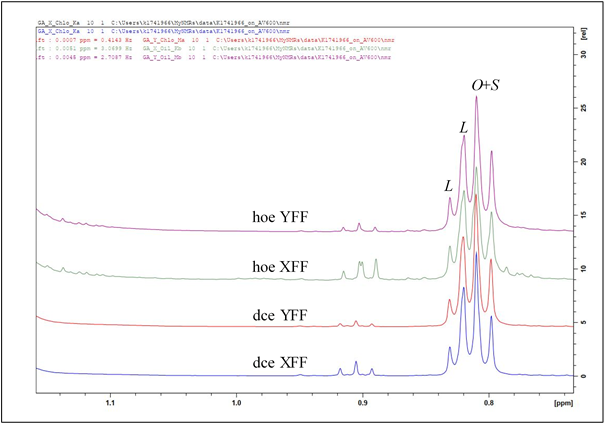

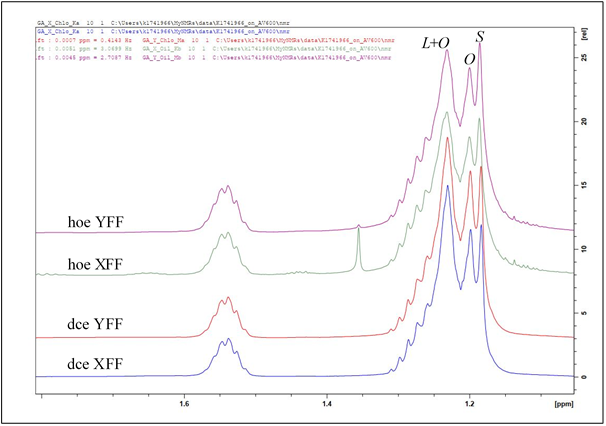

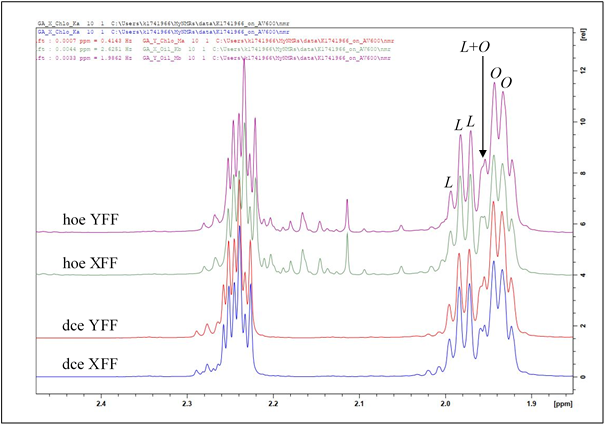

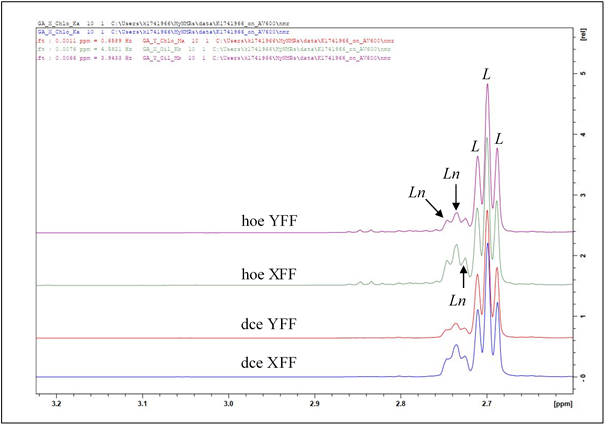

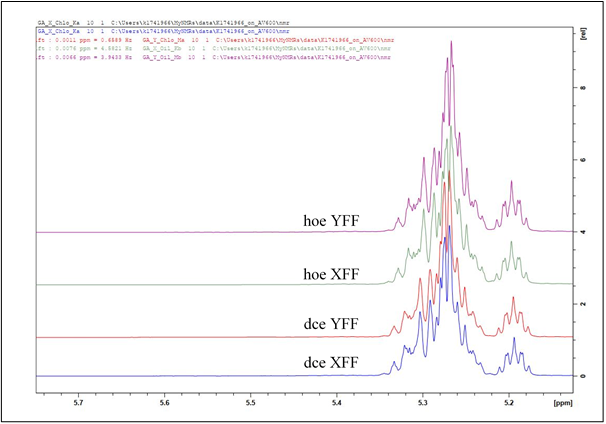


**A**

**B**

**C**

**E**

**G**

**H**

**J**

**K**

**(ii)**

**Figure S.5.** (*Continued*) ^1^H NMR spectra showing (i) major acylglycerol functions, and (ii) expanded regions of resonances A, B, C, E, G, H, J and K present within the 0.0–5.4 ppm regions of deuterochloroform and *n*-hexane-oil extracts of FF samples purchased from restaurants X and Y. Abbreviations: XFF and YFF, Restaurant X and Y French fries respectively; *Ln*, Linolenoylglycerol signal; *L*, Linoleoylglycerol signal; *O*, oleoylglycerol signal; *S*, saturated fatty acid acylglycerol signal; dce, Deuterated chloroform extract; hoe, *n*-Hexane-oil extract. Letter assignments of resonances correspond to those provided in Table 1 described in the main manuscript.


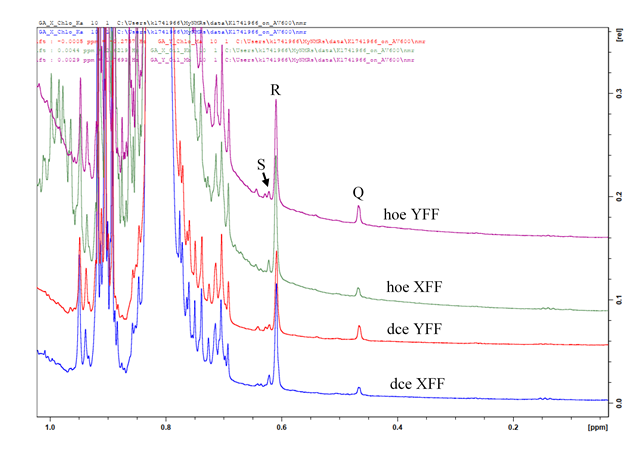

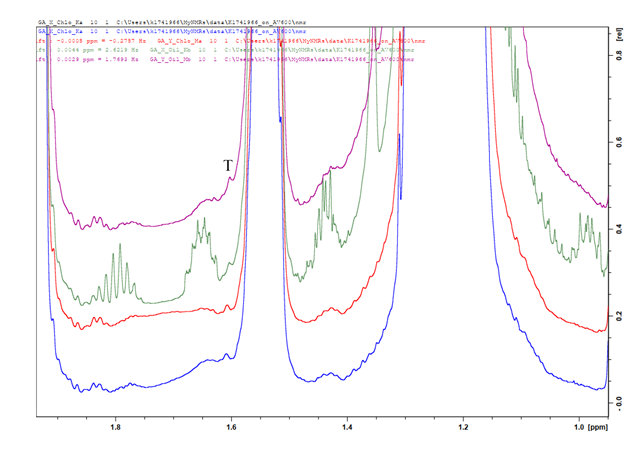

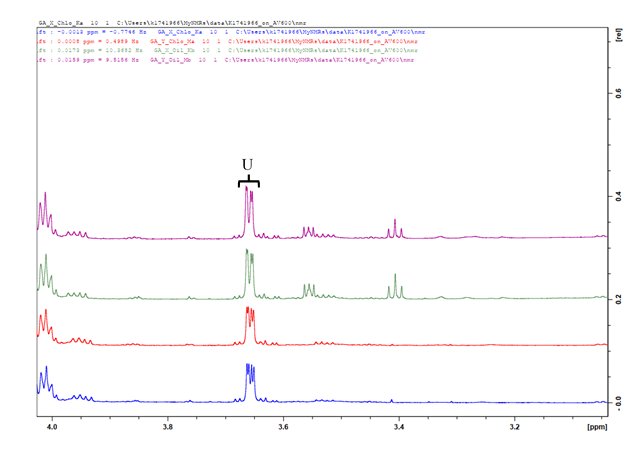


**Figure S.6.** ^1^H NMR spectra showing minor compounds present within the 0.0–3.7 ppm regions of deuterochloroform and *n*-hexane-oil extracts of FF samples purchased from restaurants X and Y. Abbreviations: XFF and YFF, and Restaurant X and Y French fries respectively; dce, Deuterochloroform extract; hoe, *n*-Hexane-oil extract. Letter assignments of resonances correspond to those provided in Table 2 of the main manuscript.

SI.4.2 Molar percentages of acyl groups and iodine value

SI.4.2.1 Acyl groups

Overall, the molar % content order of acyl groups were UFAs > oleic acid > PUFA > linoleic acid > SFA > omega-3 fatty acids > linolenic acid (Figure S.7).

**(i)**

**(ii)**


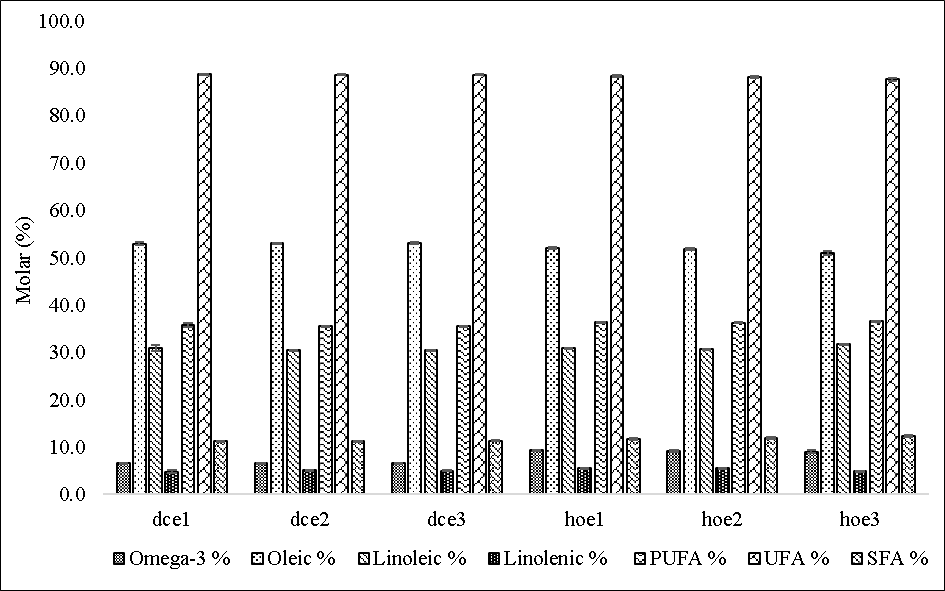

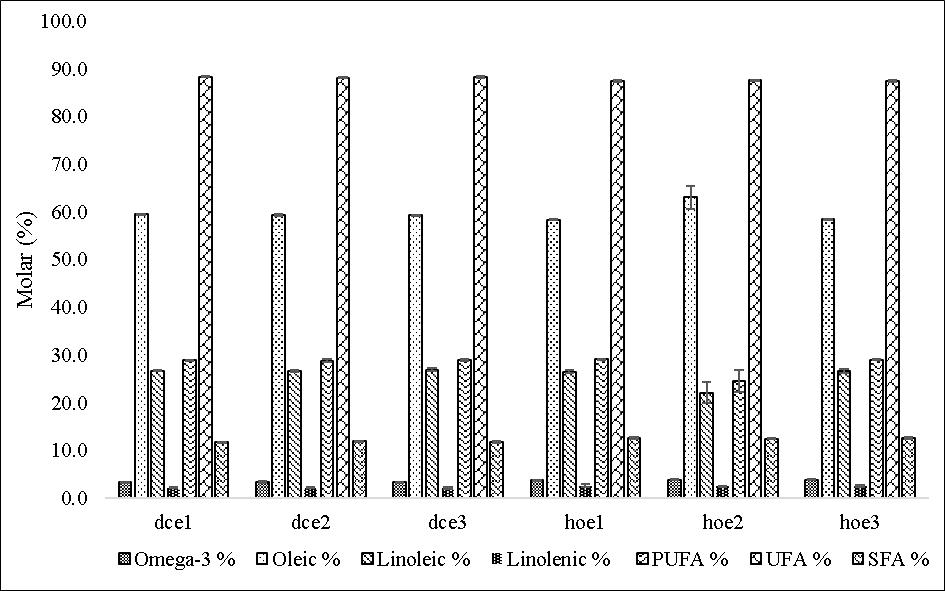


**Figure S.7.** Concentrations of acyl groups of FF culinary frying oils extracted using the deuterochloroform- and *n*-hexane methods; FF samples were purchased from restaurants X (i) and Y (ii). All values are presented as mean±SD values.

SI.4.2.2 Iodine value

The degree of unsaturation [expressed as Iodine value (IV)] of the extracts was somewhat higher in dce than in the hoe samples (Figure S.8). The differences observed between the degree of unsaturation of the XFF and YFF samples may be an indication that YFF has undergone a longer heating period, and therefore undergone a more extensive thermo-oxidation process than XFF.

**Figure S.8.** Iodine values (unit) of culinary oils extracted from FF samples purchased from restaurants X and Y. All values are presented as mean±SD values.

SI.5 Characterisation of LOPs

SI.5.1 Primary conjugated hydroperoxydiene and hydroxydiene, and hydroxymonoene LOPs

The intensities of the ^1^H NMR signals of primary LOPs varied between dce and hoe extracts of XFF and YFF. As shown on Figure S.9, hoe of XFF and YFF samples yielded higher intensities, and therefore greater concentrations of conjugated hydroperoxydienes and hydroperoxymonoenes and olefinic resonances of α,β-unsaturated aldehydes (*α*,*β*UAs). However, this is simply ascribable to the higher levels of both lipids and LOPs in the hoe extract analysis solutions. As expected, the formation of primary LOPs always preceded the evolution of secondary LOPs (Figure S.9).


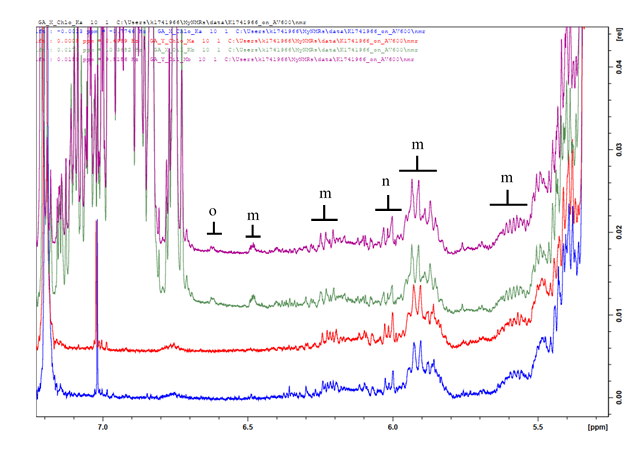

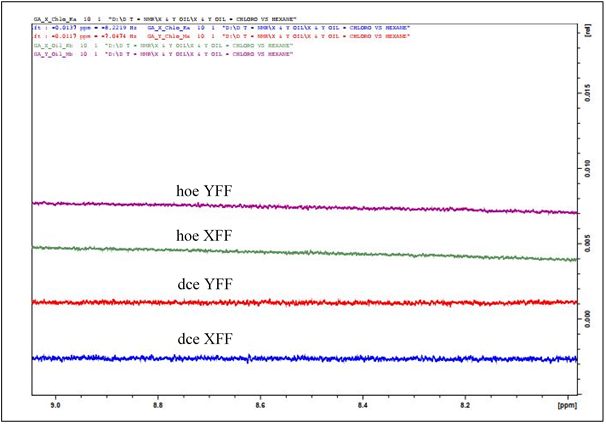


**Figure S.9.** ^1^H NMR spectra showing expanded 5.5–7.1 ppm regions of the ^1^H NMR profiles of XFF and YFF samples, both deuterochloroform and *n*-hexane-oil extracts. These spectra contain resonances assignable to conjugated diene hydroperoxydienes and hydroperoxymonoenes (both primary LOPs), and the olefinic resonances of α,β-unsaturated aldehydes. Broad resonances attributable to hydroperoxide groups (primary LOPs) which are usually present in the 8.0–8.6 ppm regions of culinary oils extracted from these FF samples were, however, either absent or obscured in these ^1^H NMR profiles. Abbreviations: XFF and YFF, and Restaurant X and Y FFs respectively; dce, Deuterated chloroform extract; hoe, *n*-Hexane-oil extract. Letter assignments of signal resonances correspond to those provided in Table S.2.

SI.5.2 Secondary LOPs

The trend observed for secondary LOPs quantified in dce and hoe extracts of both XFF and YFF samples was similar to that observed for conjugated diene hydroperoxydienes and hydroperoxymonoenes, and the olefinic resonances of α,β-unsaturated aldehydes. Epoxide resonances were more prominent in hoe extracts of XFF and YFF samples (Figure S.10), again an expected observation. An inspection of aldehydic LOP signal resonances in dce and hoe extracts of XFF and YFF (Figure S.11) showed consistencies with the aldehydic spectra of FF samples originally profiled in XFF and YFF oil extracts (Figure S.4). Distinctly, 4,5-epoxy-(E)-alkenals (signal c), 4-hydroxy-(E)-2-alkenals (signal d), and 4-oxoalkanals (signal g) were absent (or present in only trace amounts) in XFF extracts) (Figure S.11). Nonetheless, both the dce and hoe extracts of YFF showed resonances for signals c, d, and g, although a lower intensity was observed in dce extracts of YFF samples, as expected (Figure S.11).


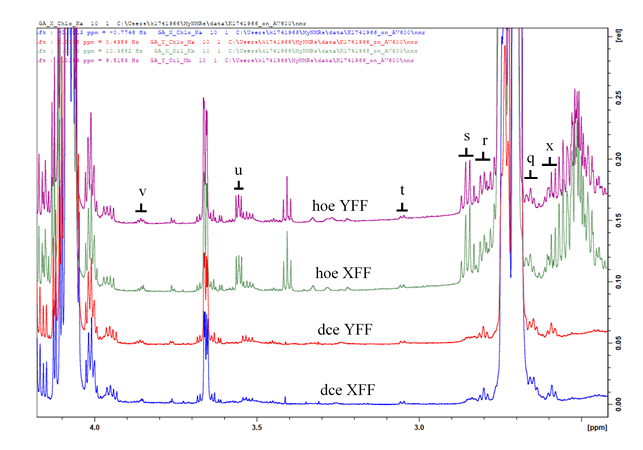


**Figure S.10.** Expanded 2.4–4.1 ppm regions of the ^1^H NMR spectra of deuterochloroform and *n*-hexane-oil extracts obtained from FF samples purchased from restaurants X and Y, showing resonances ascribable to epoxides and primary alcohols. Abbreviations: XFF and YFF, and Restaurant X and Y French fry samples respectively; dce, Deuterated chloroform extract; hoe, *n*-Hexane-oil extract. Letter assignments of resonances correspond to those provided in Table S.3.


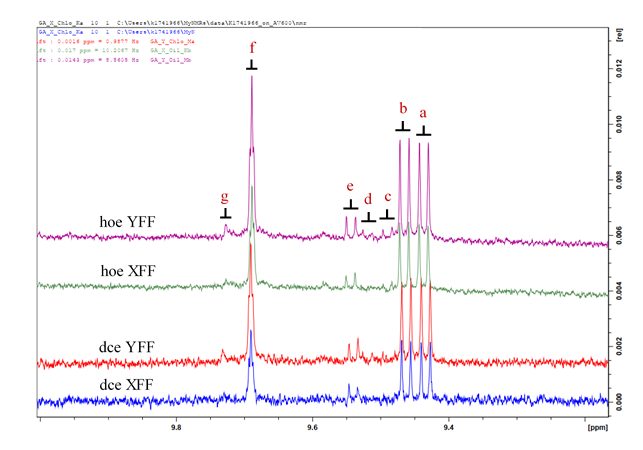


**Figure S.11.** ^1^H NMR spectra showing aldehydes (secondary LOPs) present within the 9.3–10.0 ppm regions of deuterated chloroform and *n*-hexane-oil extracts of FFs. Abbreviations: XFF and YFF, Restaurant X and Y French fries respectively; dce, deuterochloroform extract; hoe, *n*-Hexane-oil extract. Letter assignments of resonances correspond to those provided in Table S.4.

The major reason for differences observed in resonance intensities between the two extraction methods employed is that the *n*-Hexane process extracted ‘neat’ oils which was processed further for ^1^H NMR analysis (Figure 2 of the main manuscript). The deuterochloroform (C^2^HCl_3_) extraction process, however, featured the direct extraction of *ca.* 1 g masses of FFs, estimated to yield 8–17% (w/w) total lipid contents (Figure S.12).

**(i)**

**(ii)**


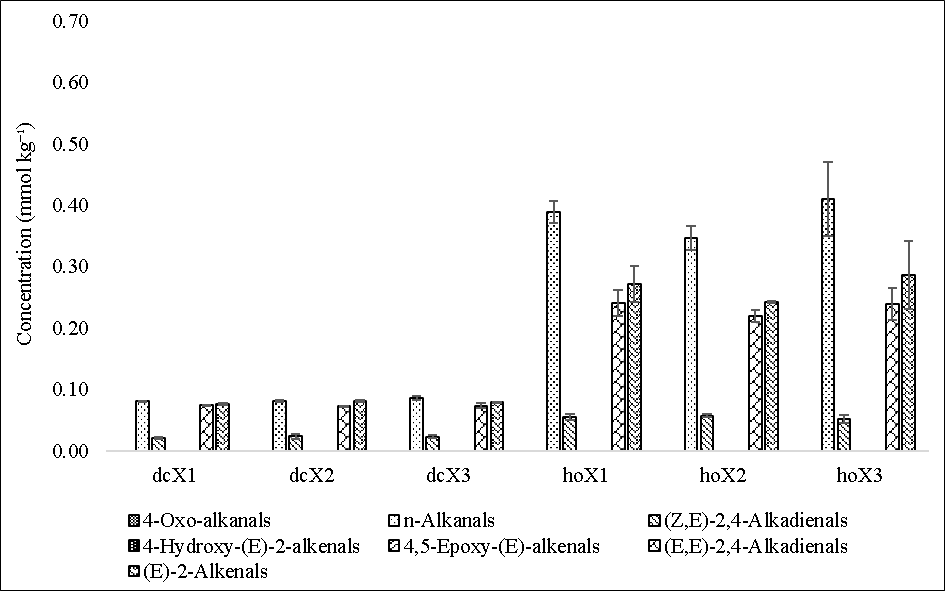

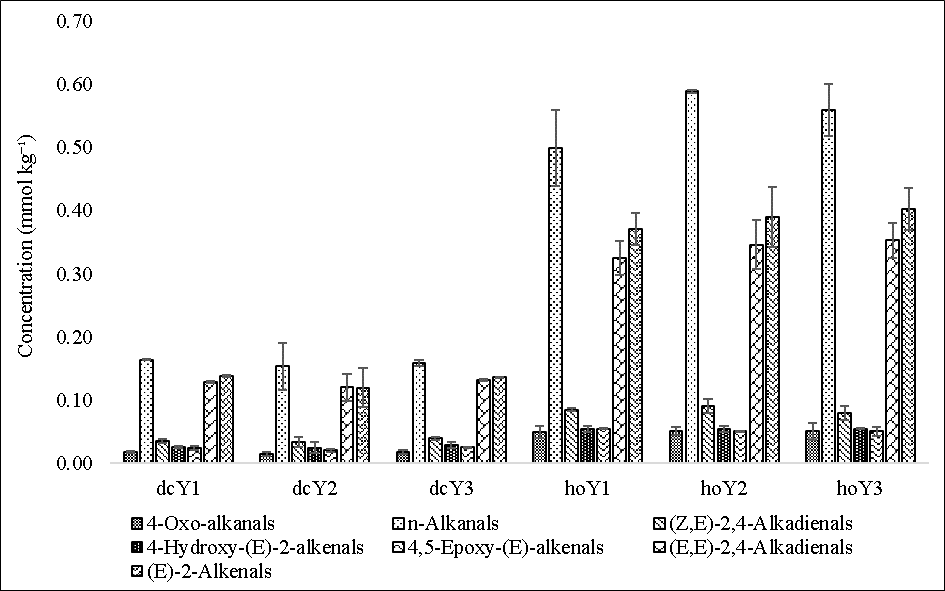


**Figure S.12.** Concentrations of secondary aldehydic LOPs of culinary frying oils which were deuterochloroform- and *n*-hexane-extracted from FF samples purchased from restaurants X (i) and Y (ii). All values are presented as mean±SD values.

SI.6 Analytical comparisons of methods utilised to extract aldehydes from FF samples

Table S.6 shows the necessary steps taken to determine the mean concentrations of LOPs for both C^2^HCl_3_ extracts and *n*-hexane oil extracts of restaurant X and Y FF samples. As noted in the main manuscript (section 3.4), normalising all aldehydic LOP values to total extracted acylglycrols was necessary to ascertain any variation that may arise from the two extraction methods employed in the study.

| LOPs | Restaurant | ^1^H NMR integral values | | Terminal -CH_3_ equivalence | | mmol LOPs/mol FA | | Average mmol LOPs/mol FA | |
| --- | --- | --- | --- | --- | --- | --- | --- | --- | --- |
|  |  | Hexane-oil extract | Deuterated chloroform extract | Hexane-oil extract | Deuterated chloroform extract | Hexane-oil extract | Deuterated chloroform extract | Hexane-oil extract | Deuterated chloroform extract |
| (*E*)-2-Alkenals | XFF | 0.1179 | 0.1262 | 0.4498 | 0.4053 | 0.26 | 0.31 | 0.24±0.03 | 0.30±0.02 |
|  |  | 0.0999 | 0.1044 | 0.4497 | 0.3654 | 0.22 | 0.29 |  |  |
|  |  | 0.1110 | 0.1135 | 0.4511 | 0.3657 | 0.25 | 0.31 | 0.24±0.01 | 0.32±0.02 |
|  |  | 0.1067 | 0.1237 | 0.4560 | 0.3656 | 0.23 | 0.34 |  |  |
|  |  | 0.1037 | 0.1146 | 0.4560 | 0.3641 | 0.23 | 0.31 | 0.24±0.02 | 0.31±0.01 |
|  |  | 0.1149 | 0.1107 | 0.4448 | 0.3645 | 0.26 | 0.30 |  |  |
|  | YFF | 0.1592 | 0.1801 | 0.4309 | 0.4143 | 0.37 | 0.43 | 0.38±0.01 | 0.43±0.00 |
|  |  | 0.1629 | 0.1793 | 0.4270 | 0.4170 | 0.38 | 0.43 |  |  |
|  |  | 0.1665 | 0.1723 | 0.4269 | 0.4151 | 0.39 | 0.42 | 0.38±0.01 | 0.41±0.00 |
|  |  | 0.1583 | 0.1572 | 0.4292 | 0.3852 | 0.37 | 0.41 |  |  |
|  |  | 0.1558 | 0.1826 | 0.4291 | 0.4168 | 0.36 | 0.44 | 0.38±0.02 | 0.43±0.01 |
|  |  | 0.1664 | 0.1757 | 0.4252 | 0.4152 | 0.39 | 0.42 |  |  |

**Table S.6** Aldehydic LOP concentrations determined from normalisation to the terminal–C**H**_3_ functions of deuterochloroform- and *n*-hexane-oil extracts of XFF and YFF samples.

| LOPs | Restaurant | ^1^H NMR integral values | | Terminal -CH_3_ equivalence | | mmol LOPs/mol FA | | Average mmol LOPs/mol FA | |
| --- | --- | --- | --- | --- | --- | --- | --- | --- | --- |
|  |  | Hexane-oil extract | Deuterated chloroform extract | Hexane-oil extract | Deuterated chloroform extract | Hexane-oil extract | Deuterated chloroform extract | Hexane-oil extract | Deuterated chloroform extract |
| (*E*,*E*)-2,4-Alkadienals | XFF | 0.1045 | 0.1214 | 0.4498 | 0.4053 | 0.23 | 0.30 | 0.22±0.02 | 0.29±0.01 |
|  |  | 0.0898 | 0.1038 | 0.4497 | 0.3654 | 0.20 | 0.28 |  |  |
|  |  | 0.1033 | 0.1025 | 0.4511 | 0.3657 | 0.23 | 0.28 | 0.22±0.02 | 0.29±0.01 |
|  |  | 0.0947 | 0.1075 | 0.4560 | 0.3656 | 0.21 | 0.29 |  |  |
|  |  | 0.0924 | 0.1089 | 0.4560 | 0.3641 | 0.20 | 0.30 | 0.20±0.00 | 0.29±0.01 |
|  |  | 0.0908 | 0.1021 | 0.4448 | 0.3645 | 0.20 | 0.28 |  |  |
|  | YFF | 0.1399 | 0.1661 | 0.4309 | 0.4143 | 0.32 | 0.40 | 0.33±0.01 | 0.41±0.02 |
|  |  | 0.1440 | 0.1763 | 0.4270 | 0.4170 | 0.34 | 0.42 |  |  |
|  |  | 0.1428 | 0.1687 | 0.4269 | 0.4151 | 0.33 | 0.41 | 0.33±0.01 | 0.42±0.02 |
|  |  | 0.1376 | 0.1652 | 0.4292 | 0.3852 | 0.32 | 0.43 |  |  |
|  |  | 0.1408 | 0.1844 | 0.4291 | 0.4168 | 0.33 | 0.44 | 0.33±0.01 | 0.43±0.02 |
|  |  | 0.1431 | 0.1729 | 0.4252 | 0.4152 | 0.34 | 0.42 |  |  |

**Table S.6** (*Continued*) Aldehydic LOP concentrations determined from normalisation to the terminal–C**H**_3_ functions of deuterochloroform- and *n*-hexane-oil extracts of XFF and YFF samples.

| LOPs | Restaurant | ^1^H NMR integral values | | Terminal -CH_3_ equivalence | | mmol LOPs/mol FA | | Average mmol LOPs/mol FA | |
| --- | --- | --- | --- | --- | --- | --- | --- | --- | --- |
|  |  | Hexane-oil extract | Deuterated chloroform extract | Hexane-oil extract | Deuterated chloroform extract | Hexane-oil extract | Deuterated chloroform extract | Hexane-oil extract | Deuterated chloroform extract |
| 4,5-Epoxy-(*E*)-alkenals | XFF | - | - | - | - | - | - | - | - |
|  |  | - | - | - | - | - | - |  |  |
|  |  | - | - | - | - | - | - | - | - |
|  |  | - | - | - | - | - | - |  |  |
|  |  | - | - | - | - | - | - | - | - |
|  |  | - | - | - | - | - | - |  |  |
|  | YFF | 0.0246 | 0.0325 | 0.4309 | 0.4143 | 0.06 | 0.08 | 0.06±0.01 | 0.08±0.00 |
|  |  | 0.0277 | 0.0309 | 0.4270 | 0.4170 | 0.06 | 0.07 |  |  |
|  |  | 0.0232 | 0.0281 | 0.4269 | 0.4151 | 0.05 | 0.07 | 0.06±0.00 | 0.07±0.00 |
|  |  | 0.0248 | 0.0250 | 0.4292 | 0.3852 | 0.06 | 0.06 |  |  |
|  |  | 0.0176 | 0.0366 | 0.4291 | 0.4168 | 0.04 | 0.09 | 0.06±0.02 | 0.08±0.00 |
|  |  | 0.0298 | 0.0338 | 0.4252 | 0.4152 | 0.07 | 0.08 |  |  |

**Table S.6** (*Continued*) Aldehydic LOP concentrations determined from normalisation to the terminal–C**H**_3_ functions of deuterochloroform- and *n*-hexane-oil extracts of XFF and YFF samples.

| LOPs | Restaurant | ^1^H NMR integral values | | Terminal -CH_3_ equivalence | | mmol LOPs/mol FA | | Average mmol LOPs/mol FA | |
| --- | --- | --- | --- | --- | --- | --- | --- | --- | --- |
|  |  | Hexane-oil extract | Deuterated chloroform extract | Hexane-oil extract | Deuterated chloroform extract | Hexane-oil extract | Deuterated chloroform extract | Hexane-oil extract | Deuterated chloroform extract |
| 4-Hydroxy-(*E*)-2-alkenals | XFF | - | - | - | - | - | - | - | - |
|  |  | - | - | - | - | - | - |  |  |
|  |  | - | - | - | - | - | - | - | - |
|  |  | - | - | - | - | - | - |  |  |
|  |  | - | - | - | - | - | - | - | - |
|  |  | - | - | - | - | - | - |  |  |
|  | YFF | 0.0231 | 0.0327 | 0.4309 | 0.4143 | 0.05 | 0.08 | 0.06±0.00 | 0.08±0.00 |
|  |  | 0.0257 | 0.0347 | 0.4270 | 0.4170 | 0.06 | 0.08 |  |  |
|  |  | 0.0241 | 0.0383 | 0.4269 | 0.4151 | 0.06 | 0.09 | 0.05±0.00 | 0.09±0.00 |
|  |  | 0.0215 | 0.0335 | 0.4292 | 0.3852 | 0.05 | 0.09 |  |  |
|  |  | 0.0162 | 0.0346 | 0.4291 | 0.4168 | 0.04 | 0.08 | 0.05±0.02 | 0.09±0.01 |
|  |  | 0.0294 | 0.0430 | 0.4252 | 0.4152 | 0.07 | 0.10 |  |  |

**Table S.6** (*Continued*) Aldehydic LOP concentrations determined from normalisation to the terminal–C**H**_3_ functions of deuterochloroform- and *n*-hexane-oil extracts of XFF and YFF samples.

| LOPs | Restaurant | ^1^H NMR integral values | | Terminal -CH_3_ equivalence | | mmol LOPs/mol FA | | Average mmol LOPs/mol FA | |
| --- | --- | --- | --- | --- | --- | --- | --- | --- | --- |
|  |  | Hexane-oil extract | Deuterated chloroform extract | Hexane-oil extract | Deuterated chloroform extract | Hexane-oil extract | Deuterated chloroform extract | Hexane-oil extract | Deuterated chloroform extract |
| (*Z*,*E*)-2,4-Alkadienals | XFF | 0.0367 | 0.0359 | 0.4498 | 0.4053 | 0.08 | 0.09 | 0.06±0.03 | 0.08±0.01 |
|  |  | 0.0207 | 0.0282 | 0.4497 | 0.3654 | 0.05 | 0.08 |  |  |
|  |  | 0.0272 | 0.0368 | 0.4511 | 0.3657 | 0.06 | 0.10 | 0.06±0.00 | 0.09±0.01 |
|  |  | 0.0248 | 0.0312 | 0.4560 | 0.3656 | 0.05 | 0.09 |  |  |
|  |  | 0.0199 | 0.0343 | 0.4560 | 0.3641 | 0.04 | 0.09 | 0.04±0.00 | 0.09±0.00 |
|  |  | 0.0195 | 0.0328 | 0.4448 | 0.3645 | 0.04 | 0.09 |  |  |
|  | YFF | 0.0364 | 0.0453 | 0.4309 | 0.4143 | 0.08 | 0.11 | 0.09±0.00 | 0.11±0.01 |
|  |  | 0.0388 | 0.0421 | 0.4270 | 0.4170 | 0.09 | 0.10 |  |  |
|  |  | 0.0377 | 0.0456 | 0.4269 | 0.4151 | 0.09 | 0.11 | 0.09±0.00 | 0.11±0.00 |
|  |  | 0.0375 | 0.0403 | 0.4292 | 0.3852 | 0.09 | 0.10 |  |  |
|  |  | 0.0292 | 0.0482 | 0.4291 | 0.4168 | 0.07 | 0.12 | 0.07±0.01 | 0.12±0.00 |
|  |  | 0.0337 | 0.0479 | 0.4252 | 0.4152 | 0.08 | 0.12 |  |  |

**Table S.6** (*Continued*) Aldehydic LOP concentrations determined from normalisation to the terminal–C**H**_3_ functions of deuterochloroform- and *n*-hexane-oil extracts of XFF and YFF samples.

**Table S.6** (*Continued*) Aldehydic LOP concentrations determined from normalisation to the terminal–C**H**_3_ functions of deuterochloroform- and *n*-hexane-oil extracts of XFF and YFF samples.

| LOPs | Restaurant | ^1^H NMR integral values | | Terminal -CH_3_ equivalence | | mmol LOPs/mol FA | | Average mmol LOPs/mol FA | |
| --- | --- | --- | --- | --- | --- | --- | --- | --- | --- |
|  |  | Hexane-oil extract | Deuterated chloroform extract | Hexane-oil extract | Deuterated chloroform extract | Hexane-oil extract | Deuterated chloroform extract | Hexane-oil extract | Deuterated chloroform extract |
| *n*-Alkanals | XFF | 0.1611 | 0.1305 | 0.4498 | 0.4053 | 0.36 | 0.32 | 0.35±0.02 | 0.32±0.01 |
|  |  | 0.1496 | 0.1126 | 0.4497 | 0.3654 | 0.33 | 0.31 |  |  |
|  |  | 0.1533 | 0.1102 | 0.4511 | 0.3657 | 0.34 | 0.30 | 0.35±0.02 | 0.31±0.01 |
|  |  | 0.1663 | 0.1174 | 0.4560 | 0.3656 | 0.36 | 0.32 |  |  |
|  |  | 0.1522 | 0.1207 | 0.4560 | 0.3641 | 0.33 | 0.33 | 0.35±0.02 | 0.34±0.01 |
|  |  | 0.1606 | 0.1236 | 0.4448 | 0.3645 | 0.36 | 0.34 |  |  |
|  | YFF | 0.2263 | 0.2154 | 0.4309 | 0.4143 | 0.53 | 0.52 | 0.52±0.01 | 0.52±0.00 |
|  |  | 0.2177 | 0.2142 | 0.4270 | 0.4170 | 0.51 | 0.51 |  |  |
|  |  | 0.2446 | 0.2196 | 0.4269 | 0.4151 | 0.57 | 0.53 | 0.58±0.01 | 0.53±0.00 |
|  |  | 0.2499 | 0.2022 | 0.4292 | 0.3852 | 0.58 | 0.52 |  |  |
|  |  | 0.2215 | 0.2102 | 0.4291 | 0.4168 | 0.52 | 0.50 | 0.52±0.01 | 0.50±0.00 |
|  |  | 0.2267 | 0.2093 | 0.4252 | 0.4152 | 0.53 | 0.50 |  |  |

| LOPs | Restaurant | ^1^H NMR integral values | | Terminal -CH_3_ equivalence | | mmol LOPs/mol FA | | Average mmol LOPs/mol FA | |
| --- | --- | --- | --- | --- | --- | --- | --- | --- | --- |
|  |  | Hexane-oil extract | Deuterated chloroform extract | Hexane-oil extract | Deuterated chloroform extract | Hexane-oil extract | Deuterated chloroform extract | Hexane-oil extract | Deuterated chloroform extract |
| 4-Oxo-alkanals | XFF | - | - | - | - | - | - | - | - |
|  |  | - | - | - | - | - | - |  |  |
|  |  | - | - | - | - | - | - | - | - |
|  |  | - | - | - | - | - | - |  |  |
|  |  | - | - | - | - | - | - | - | - |
|  |  | - | - | - | - | - | - |  |  |
|  | YFF | 0.0228 | 0.0221 | 0.4309 | 0.4143 | 0.05 | 0.05 | 0.06±0.01 | 0.06±0.00 |
|  |  | 0.0279 | 0.0246 | 0.4270 | 0.4170 | 0.07 | 0.06 |  |  |
|  |  | 0.0257 | 0.0215 | 0.4269 | 0.4151 | 0.06 | 0.05 | 0.05±0.01 | 0.05±0.00 |
|  |  | 0.0202 | 0.0204 | 0.4292 | 0.3852 | 0.05 | 0.05 |  |  |
|  |  | 0.0174 | 0.0227 | 0.4291 | 0.4168 | 0.04 | 0.05 | 0.05±0.02 | 0.06±0.01 |
|  |  | 0.0283 | 0.0258 | 0.4252 | 0.4152 | 0.07 | 0.06 |  |  |

**Table S.6** (*Continued*) Aldehydic LOP concentrations determined from normalisation to the terminal–C**H**_3_ functions of deuterochloroform- and *n*-hexane-oil extracts of XFF and YFF samples.


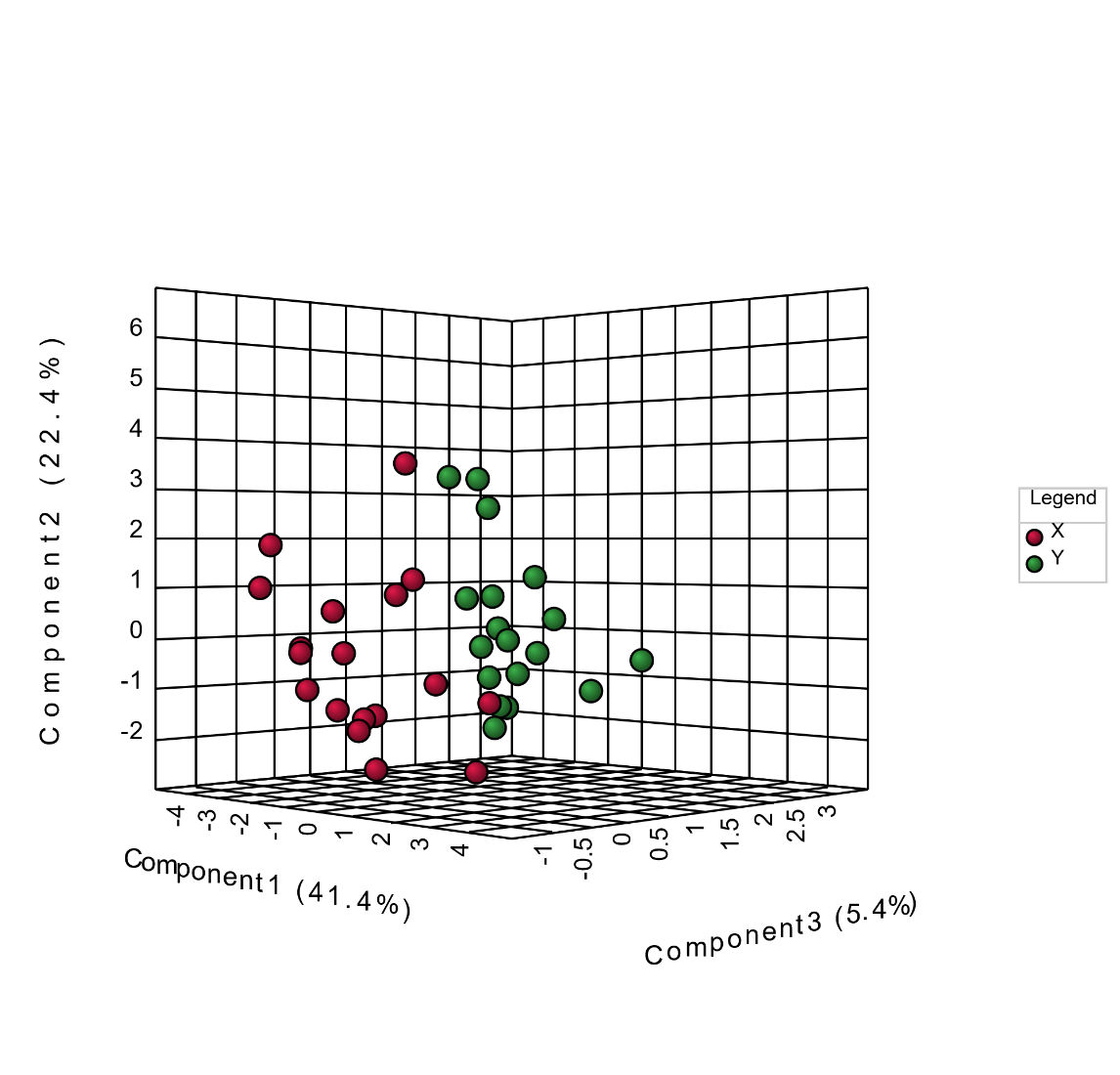


**Figure S.13.** Three-dimensional PLS-DA component 3 versus component 2 versus component 1 scores plot showing a high level of distinctiveness between the trace metal concentration patterns of extracted oil samples from fast-food restaurants X and Y. Al, Cu, Fe, Mn, Pb and V loaded significantly on component 1; Ba, Ni, Ti and Zn loaded on component 2; and marginally Cr and Cd loaded on component 3.

SI.7 ICP-OES profiles of trace metals in oil extracts of XFF and YFF samples

When expressed relative to the weight of FFs to *n*-hexane-aided oil extraction, Table S.7 highlights the portions of FFs that would have to be ingested, taking into consideration an average body weight of 77.5 kg, to meet the daily requirement of the trace metals set by European Population Reference Intake (PRI), Food and Nutrition Board (FNB), and European Food Safety Authority (EFSA). This has been discussed in section 4.4 of the main manuscript.

**Table S.7** Portions (1 portion = 30 g FF) of FF samples from restaurants X and Y that meet the daily requirement of trace metals

|  | Trace metals | | | | | | | | | | | |
| --- | --- | --- | --- | --- | --- | --- | --- | --- | --- | --- | --- | --- |
| Duration | Al | Ba | Cd | Cr | Cu | Fe | Mn | Ni | Pb | Ti | V | Zn |
|  | Restaurant X French fries (XFF) | | | | | | | | | | | |
| Friday Morning | 43.51 | 566.22 | 635.35 | 44419.70 | 1408.81 | 703.88 | 9497.99 | <LOQ | 783.80 | N/A | 24178.71 | 974.24 |
| Friday Afternoon | 32.98 | 345.00 | 551.92 | 47130.45 | 905.14 | 202.19 | 5611.71 | <LOQ | 580.91 | N/A | 17498.72 | 807.63 |
| Friday Evening | 24.92 | 332.37 | 258.62 | 23832.18 | 774.01 | 203.97 | 4755.11 | <LOQ | 370.26 | N/A | 36716.55 | 714.04 |
| **Daily Average** | **33.80** | **414.53** | **481.96** | **38460.78** | **1029.32** | **370.02** | **6621.60** | **<LOQ** | **578.32** | **N/A** | **26131.33** | **831.97** |
|  |  |  |  |  |  |  |  |  |  |  |  |  |
| Saturday Morning | 44.08 | 586.76 | 767.11 | 52626.19 | 1623.49 | 827.56 | 10319.60 | <LOQ | 337.20 | N/A | 53305.01 | 1471.56 |
| Saturday Afternoon | 56.27 | 772.41 | 615.78 | 72051.93 | 2057.42 | 1115.43 | 13875.59 | <LOQ | 1061.90 | N/A | 36368.27 | 3957.90 |
| Saturday Evening | 46.92 | 610.65 | 1645.39 | 61164.14 | 1991.87 | 936.82 | 11718.24 | 995.42 | 865.77 | N/A | 73389.48 | 3020.16 |
| **Daily Average** | **49.09** | **656.61** | **1009.43** | **61947.42** | **1890.93** | **959.94** | **11971.14** | **995.42** | **754.95** | **N/A** | **54354.26** | **2816.54** |
|  |  |  |  |  |  |  |  |  |  |  |  |  |
| Monday Morning | 36.25 | 509.58 | 509.82 | 29715.39 | 1120.27 | 590.41 | 8488.00 | 343.48 | 340.10 | N/A | 94689.52 | 1850.78 |
| Monday Afternoon | 45.99 | 553.50 | 980.95 | 40010.82 | 1477.96 | 710.63 | 9619.93 | <LOQ | 933.19 | N/A | 30860.90 | 1434.56 |
| Monday Evening | 156.71 | 454.70 | 424.71 | 43308.60 | 1840.47 | 949.84 | 19328.14 | <LOQ | 1179.50 | N/A | <LOQ | 1515.44 |
| **Daily Average** | **79.65** | **505.93** | **638.49** | **37678.27** | **1479.57** | **750.29** | **12478.69** | **343.48** | **817.60** | **N/A** | **62775.21** | **1600.26** |
|  |  |  |  |  |  |  |  |  |  |  |  |  |
| **Overall Average** | **54.18** | **525.69** | **709.96** | **46028.82** | **1466.60** | **693.41** | **10357.14** | **669.45** | **716.96** | **N/A** | **47753.60** | **1749.59** |
|  |  |  |  |  |  |  |  |  |  |  |  |  |
| *Daily maximum legal limit of trace metals (µg) per average body weight (77.5 kg) | 11100^EFSA^ | 15500^EFSA^ | 190 ^EFSA^ | 23250^EFSA^ | 1100^PRI^ | 9000^FNB^ | 5500^EFSA^ | 217^EFSA^ | 1940^EFSA^ | - | 1800^FNB^ | 8250^PRI^ |

Abbreviations: Aluminium (Al), Barium (Ba), Cadmium (Cd), Chromium (Cr), Copper (Cu), Iron (Fe), Manganese (Mn), Nickel (Ni), Lead (Pb), Titanium (Ti), Vanadium (V) and Zinc (Zn). Less than limit of quantification (<LOQ). European Population Reference Intake (PRI), Food and Nutrition Board (FNB), European Food Safety Authority (EFSA), Not Applicable (N/A), *Values are derived values per day.

**Table S.7** (*Continued*) Portions (1 portion = 30 g FF) of FF samples from restaurants X and Y that meet the daily requirement of trace metals

|  | Trace metals | | | | | | | | | | | |
| --- | --- | --- | --- | --- | --- | --- | --- | --- | --- | --- | --- | --- |
| Duration | Al | Ba | Cd | Cr | Cu | Fe | Mn | Ni | Pb | Ti | V | Zn |
|  | Restaurant Y French fries (YFF) | | | | | | | | | | | |
| Friday Morning | 73.96 | 212.08 | 262.20 | 17929.76 | 710.50 | 380.51 | 7565.08 | <LOQ | 495.82 | N/A | <LOQ | 344.57 |
| Friday Afternoon | 103.34 | 300.19 | 394.27 | 21639.95 | 1204.62 | 584.46 | 12912.24 | <LOQ | 768.94 | N/A | <LOQ | 1520.55 |
| Friday Evening | 82.32 | 253.30 | 332.70 | 29342.69 | 834.25 | 488.51 | 10543.97 | <LOQ | 629.97 | N/A | <LOQ | 809.67 |
| **Daily Average** | **86.54** | **255.19** | **329.72** | **22970.80** | **916.46** | **484.49** | **10340.43** | **<LOQ** | **631.58** | **N/A** | **<LOQ** | **891.59** |
|  |  |  |  |  |  |  |  |  |  |  |  |  |
| Saturday Morning | 73.73 | 227.15 | 281.41 | 23618.00 | 851.78 | 579.04 | 11288.30 | <LOQ | 573.26 | N/A | <LOQ | 488.20 |
| Saturday Afternoon | 70.55 | 213.19 | 257.97 | 23268.78 | 825.36 | 589.95 | 10522.14 | <LOQ | 690.43 | N/A | <LOQ | 870.97 |
| Saturday Evening | 75.10 | 230.39 | 207.94 | 15363.88 | 728.52 | 459.60 | 9739.68 | <LOQ | 711.53 | N/A | <LOQ | 974.58 |
| **Daily Average** | **73.13** | **223.58** | **249.11** | **20750.22** | **801.89** | **542.86** | **10516.71** | **<LOQ** | **658.41** | **N/A** | **<LOQ** | **777.91** |
|  |  |  |  |  |  |  |  |  |  |  |  |  |
| Monday Morning | 64.55 | 206.52 | 202.89 | 18858.47 | 606.40 | 429.20 | 8676.03 | <LOQ | 297.52 | N/A | <LOQ | 364.07 |
| Monday Afternoon | 60.76 | 192.03 | 119.88 | 17801.84 | 557.26 | 454.01 | 11280.12 | <LOQ | 564.81 | N/A | <LOQ | 394.40 |
| Monday Evening | 57.14 | 182.96 | 243.40 | 16856.25 | 653.26 | 543.85 | 12178.91 | 419.83 | 1025.12 | N/A | <LOQ | 702.97 |
| **Daily Average** | **60.82** | **193.84** | **188.72** | **17838.85** | **605.64** | **475.68** | **10711.69** | **419.83** | **629.15** | N/A | **<LOQ** | **487.15** |
|  |  |  |  |  |  |  |  |  |  |  |  |  |
| **Overall Average** | **73.50** | **224.20** | **255.85** | **20519.96** | **774.66** | **501.01** | **10522.94** | **419.83** | **639.71** | **N/A** | **<LOQ** | **718.88** |
|  |  |  |  |  |  |  |  |  |  |  |  |  |
| Daily maximum legal limit of trace metals (µg) per average body weight (77.5 kg) | 11100^EFSA^ | 15500^EFSA^ | 190 ^EFSA^ | 23250^EFSA^ | 1100^PRI^ | 9000^FNB^ | 5500^EFSA^ | 217^EFSA^ | 1940^EFSA^ | - | 1800^FNB^ | 8250^PRI^ |

Abbreviations: Aluminium (Al), Barium (Ba), Cadmium (Cd), Chromium (Cr), Copper (Cu), Iron (Fe), Manganese (Mn), Nickel (Ni), Lead (Pb), Titanium (Ti), Vanadium (V) and Zinc (Zn). Less than limit of quantification (<LOQ). European Population Reference Intake (PRI), Food and Nutrition Board (FNB), European Food Safety Authority (EFSA), Not Applicable (N/A),

Reference

S1. Goicoechea, E., and Guillén, M. D. (2010). Analysis of hydroperoxides, aldehydes and epoxides by ^1^H Nuclear Magnetic Resonance in sunflower oil oxidized at 70 and 100°C. J. Agric. and Food Chem. 58:10, 6234–6245.

S2. Guillén, M. D., and Ruiz, A. (2003). Rapid simultaneous determination by proton NMR of unsaturation and composition of acyl groups in vegetable oils. Eur. J. Lipid Sci. Tech*.* 105:11, 688–696.

S3. Guillén, M. D., and Uriarte, P. S. (2012). Study by ^1^H NMR spectroscopy of the evolution of extra virgin olive oil composition submitted to frying temperature in an industrial fryer for a prolonged period of time. Food Chem*.* 134:1, 162–172.

S4. Le Gresley, A., Ampem, G., Grootveld, M., Percival, B., Naughton, D. P. (2019). Characterisation of peroxidation products arising from culinary oils exposed to continuous and discontinuous thermal degradation processes. Food Funct. 10: 7952–7966.

S5. Markaverich, B. M. *et al*. (2005). Leukotoxins diols from ground corncob bedding disrupt estrus cyclicity in rats and stimulate MCF-7 breast cancer cell proliferation. Environ. Health Perspect. 113:12, 1698–1704.

S6. Martínez-Yusta, A., Goicoechea, E., and Guillén, M. D. (2014). A review of thermo-oxidative degradation of food lipids studied by ^1^H NMR spectroscopy: influence of degradative conditions and food lipid nature. Compr. Rev. Food Sci. Food Saf*.* 13:5, 838–859.

S7. Thompson, D. A., and Hammock, B. D. (2007). Dihydroxyoctadecamonoenoate esters inhibit the neutrophil respiratory burst. J. Biosci. 32:2, 279–291.
